# Reshuffling the global R&D deck, 1980-2050

**DOI:** 10.1371/journal.pone.0213801

**Published:** 2019-03-29

**Authors:** Steven P. Dehmer, Philip G. Pardey, Jason M. Beddow, Yuan Chai

**Affiliations:** 1 HealthPartners Institute, Minneapolis, Minnesota, United States of America; 2 Department of Applied Economics, University of Minnesota, St. Paul, United States of America; International Maize and Wheat Improvement Center (CIMMYT), MEXICO

## Abstract

Based on more recent science spending developments in countries such as China, Korea, India and Brazil, there is a growing sense that the world’s scientific deck of cards is in the midst of a major reshuffle. But it is not clear if this reordering is limited to just the top spenders, or, indeed, how these changes have been playing out over the longer term. The new, more comprehensive research and development (R&D) spending estimates presented and discussed here reveal that we are in the midst of a possibly game-changing, albeit partial and perhaps irregular, reshuffle of the global R&D deck. These changes have potentially profound domestic and international economic development implications over the medium to long term. Notably, the fortunes of many of the world’s poorer countries continue to look bleak. Using the evolving structure of past R&D spending to project forward, and absent marked changes in science policies and spending priorities, we foresee a continuing and substantial shift in the geography of R&D towards parts of Asia, along with a continuing large, and in many respects growing, gap between the world’s scientific haves and have-nots.

## Introduction

R&D is a risky but calculated endeavor, like many games of cards. But unlike these games, R&D gives society the advantage, with a sizable positive payout on balance [1, 2]. Spreading risk and return, R&D is also an increasingly globalized endeavor. Scientists follow the money or the scientific opportunities, basing their operations accordingly [3, 4]. Firms make decisions on where in the world to locate their R&D activities, in part, with an eye to the pattern of R&D investments by other firms and public agencies [5, 6]. Cognizant of the innovation and, ultimately, the economic growth consequences of R&D, governments enact policies that affect the amount and nature of domestic investments in research and a country’s relative scientific and economic standing. Although the international interconnectedness of science is on the rise [7], information on the retrospective and prospective global patterns of investments in R&D is still scant. Here we present a substantially expanded view of the past, present, and prospective futures of global R&D spending.

## Methodology

The changing global gross domestic expenditure on research and development (GERD) landscape assessed here draws on newly developed International Science and Technology Practice and Policy center (InSTePP, University of Minnesota) estimates. The historical GERD series for the period 1980–2013 was constructed in several, interlinked steps. We first drew from a number of primary [8–12] and other sources (Table A1 in S1 File) to form a core set of harmonized annual, country-specific GERD intensities—defined as the quotient of total R&D spending to gross domestic product (GDP)—for each country-year for the period 1980–2013. From the core set of research-intensity data, a complete panel of GERD intensity ratio estimates for 199 countries covering the period 1980–2013 were derived using a number of replicable interpolation methods. Specifically, gaps in research intensities for any given country were interpolated assuming a constant rate of change in research intensities for the intervening missing years, while missing values at the beginning or end of a time series were formed by extrapolating backward or forward a constant value from the most adjacent known value. For countries with no reported GERD research-intensity data, estimates were constructed by calculating the weighted average for peer economies in each country’s geographical region (see country classifications by region in Table A2 in S1 File).

With a complete panel of GERD intensity ratio estimates in hand, we then estimated GERD by multiplying these country-specific research intensities by the corresponding annual GDP time-series (expressed in 2009 international, i.e., purchasing power parity, PPP, dollars) taken from [13]. To parse the GERD totals into their respective public (i.e., public expenditure on R&D, PERD) and private (i.e., business expenditure on R&D, BERD) components, we used the “by performer” data (i.e., based on who conducted the research, rather than by who paid for it) from the GERD intensity sources to estimate the public and private shares. For countries without such data, estimates were calculated using their corresponding regional averages. More detailed descriptions of our data sources and interpolation procedures are provided in S1 File.

These new data encompass a period that includes the collapse and subsequent gradual, but far from complete, recovery of science spending in the group of countries that constituted the Former Soviet Union. Notably, they also include estimates for many of the world’s low- and middle-income countries that have thus far been largely excluded from a comprehensive consideration of the changing pattern of global science spending. Spanning 199 countries for the period 1980–2013, the InSTePP series accounts for 6,554 country-years, of which 1,763 (27.2%) of these country-years—constituting 93.7% of the accumulated $30.5 trillion of global R&D spending (2009 purchasing power parity, PPP, dollars) for the period 1980–2013—were based on directly sourced GERD intensities (as distinct from interpolated or econometrically derived estimates). This expands upon the 61 countries for which the U.S. National Science Foundation (NSF) tabulated country-level GERD estimates for circa 2013 (Table 4–4 in [14]) and the 68 country-level estimates for NSF’s circa 2015 estimates (Table 4–5 in [15]).

The future is inherently uncertain, but based on the assumption that the empirical regularities of the past persist over the coming decades, we used our newly developed historical GERD series for the period 1980–2013 to form projections of regional and global GERD intensities to 2050. Taking advantage of the identity relationship that GERD can be exactly decomposed into the product of the intensity ratio (i.e., GERD as a percentage of GDP) and GDP, we first projected forward country-level research intensity ratios and then applied those intensity ratios to corresponding country-specific GDP projections to derive GERD projections. More specifically, country-level projections of GERD intensity ratios to 2050 were formed using a dynamic second order polynomial algorithm estimated by regressing the growth rate of the GERD intensity ratio on the GERD intensity ratio itself, thus exploiting a significant regularity in the country-level relationship we observed between the historical rate of growth in GERD intensity and the GERD intensity level (see section 2.2 in S1 File). Our intensity ratio projections were then applied to a compilation of GDP projections derived from an ensemble of GDP forecasts obtained from various sources [16–24] to project GERD for each country-year through to 2050. We derived our preferred midline GERD projections, along with high and low variants thereof, corresponding with the variation we found in the GDP projections. By applying research intensity ratio projections to GDP projections to derive GERD projections, our method has the distinct advantages of parsimony and replicability that take advantage of the cross-sectional and inter-temporal regularity in GERD intensity ratios and transparently apply a well-documented and generally robust set of GDP projections. More complete details of our prospective GERD estimates and the data and methodological underpinnings of the GERD projections are also provided in S1 File.

## Results and discussion

### The current state of global R&D

In 2013, science spending worldwide totaled $1.61 trillion in GERD (2009 PPP dollars, and same units throughout), equivalent to 1.69% of global GDP in that year (Table 1). The United States held a prominent position in terms of overall R&D spending, accounting for 27.4% ($441 billion) of the global GERD total. China ranked second with a 19.2% share ($309 billion), having edged Japan into third place in 2008. Global spending is highly concentrated; the 20 top-ranked countries accounted for 90.1% of expenditures while the bottom 100 countries accounted for just 0.4% of the global total.

**Table 1:** GERD country rankings, 1980, 2013 and 2050.

		Gross domestic expenditures on R&D		
		$b	% GDP	per capita
**1980**				
1	United States	149.5	2.32	649.4
2	USSR	80.1	2.58	302.4
3	Japan	49.3	2.19	425.0
4	Germany	42.9	2.35	542.3
5	France	24.2	1.86	449.2
6	United Kingdom	24.0	2.26	426.1
7	Italy	9.8	0.76	174.6
8	Canada	7.8	1.20	318.3
9	Netherlands	6.7	1.87	473.9
10	China	5.5	0.80	5.6
	**Global total**	**478.6**	**1.47**	**108.1**
	Top 10	399.8	2.11	212.8
	Top 20	437.2	1.81	150.9
	Bottom 100	2.3	0.26	6.1
**2013**				
1	United States	441.2	2.80	1,378.6
2	China	308.8	1.99	222.9
3	Japan	153.4	3.49	1,206.1
4	Germany	94.0	2.94	1,136.8
5	Republic of Korea	62.3	4.15	1,264.5
6	France	51.0	2.23	793.5
7	India	50.6	0.81	40.4
8	Former Soviet Republics	42.8	0.92	148.3
9	United Kingdom	36.9	1.63	585.1
10	Brazil	34.1	1.21	170.1
	**Global total**	**1,610.0**	**1.69**	225.7
	Top 10	1,275.2	2.17	332.7
	Top 20	1,450.8	2.11	350.1
	Bottom 100	6.5	0.19	9.0
**2050 (midline projection)**				
1	China	1,955.3	3.45	1,411.8
2	United States	976.5	3.45	2,436.1
3	India	848.3	2.31	523.6
4	Former Soviet Republics	296.4	2.94	1,086.9
5	Brazil	266.6	3.39	1,153.6
6	Japan	248.1	3.46	2,290.1
7	Turkey	163.7	3.05	1,730.6
8	Republic of Korea	160.3	3.46	3,140.5
9	United Kingdom	150.7	3.44	2,060.1
10	Germany	141.0	3.46	1,943.6
	**Global total**	**6,758.3**	**2.29**	710.1
	Top 10	5,206.9	3.15	1,208.3
	Top 20	6,080.7	3.15	1,270.4
	Bottom 100	19.8	0.09	12.8

Source: Authors calculations. S1 File gives primary sources for historical R&D and GDP estimates, and details on projected estimates.

Notes: GERD expenditure totals and per capita figures in 2009 PPP (purchasing power parity) units. For presentation and projections purposes, the Former Soviet Republics are an aggregate of 15 countries, including the Russian Federation, who’s GERD in 2013 was $35.1 billion. The %GDP and per capita estimates for the global total, top 10, 20 and bottom 100 represent weighted averages of the respective country aggregates.

**Fig 1:**
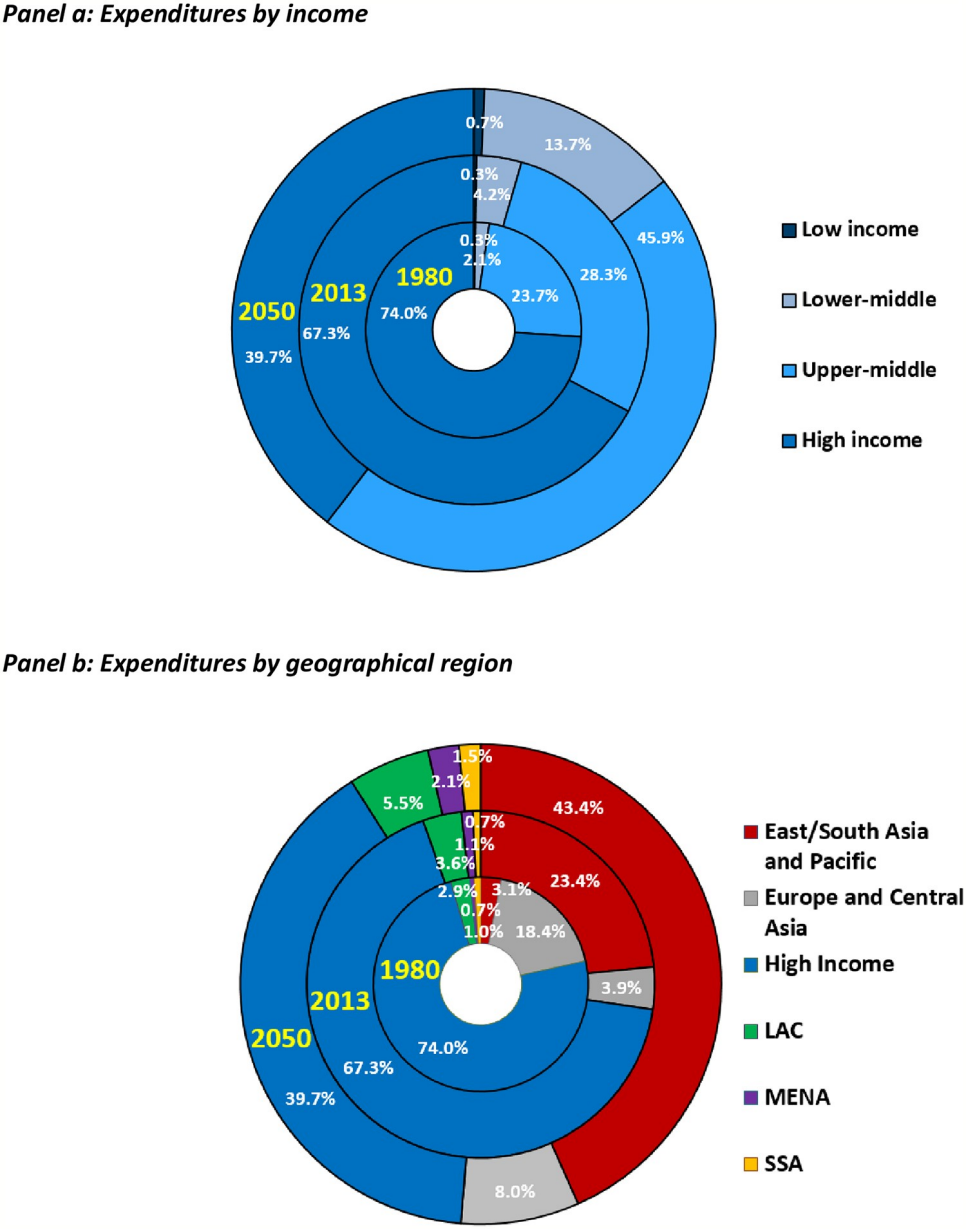
Global R&D spending totals, 1980, 2013 and 2050. Source: See Table 1. Notes: These estimates consist of total (public and private) R&D spending for 175 countries and country aggregates (based on data from 199 countries). Panel a: Countries are grouped into income classes using the 2013 World Bank schema [25]. Panel b: The regional grouping is based on a World Bank classification [25]. However, the diagram groups the countries of South Asia, East Asia and Pacific into a single East/South Asia and Pacific grouping. Europe and Central Asia includes the Former Soviet Republics, Turkey and the low- and middle-income countries of Eastern Europe. The 2050 totals reflect the baseline scenario.

The concentration of science spending can be seen by income level and geographical region as well (Fig 1 and S1 Table, based on World Bank schema [25]). The world’s rich countries accounted for just over two thirds of the global R&D performed in 2013, with 32.4% of the world’s R&D spending taking place in middle-income countries (Fig 1). Dramatically, the 33 low-income countries—home to 11.5% (823.8 million) of the planet’s 2013 population [26]—invested only $4.4 billion in R&D, just 0.3% of the global total. The capacity for science-based innovation in this part of the world is still severely limited. Among low- and middle-income countries, R&D spending was highly concentrated in East and South Asia and the Pacific, which accounted for 23.4% ($376.7 billion) of the global total in 2013. In contrast, only 3.6% ($57.8 billion) of global GERD was conducted in Latin America and the Caribbean, and a scant 0.7% ($11.9 billion) was conducted in Sub-Saharan Africa. Notably, science spending is highly concentrated within each region as well, with China accounting for 82.0%, Brazil 59.0%, and South Africa 39.6% of their respective regional totals in 2013.

Some disparities in R&D spending are to be expected, not least as countries with larger economies and populations have more capacity to support robust scientific infrastructures. Nevertheless, even among the top-ranked science spenders there can be sizable discrepancies in R&D spending even when adjusting for these factors (Table 1). The top-10 ranked R&D countries in 2013 spent an average of $2.17 on R&D for every $100 of GDP, or almost $333 per person. South Korea spent well-above the group average at $4.15 for every $100 of GDP and more than five times the share of their economy in comparison to seventh overall ranked India, which spent 81 cents on R&D for every $100 of its GDP. Second ranked overall, China’s intensity of R&D spending was close to the top-10 group average at $1.99 for every $100 of GDP, but was markedly different in terms of spending per capita, which was $223 per person—a good distance from the more than $1,296 per person spent by the other countries ranked in the top five. Still, China’s R&D spending per capita in 2013 was substantially higher than the upper-middle-income average ($169 per person), as was its GERD intensity, which averaged $1.31 per $100 of GDP among upper-middle-income countries. Likewise, India, a lower-middle-income country, spent more than its income class average on R&D relative to GDP (81 versus 49 cents per $100 of GDP in 2013) and relative to population size (around $40 versus $27 per person). In stark and sobering contrast, the bottom 100 ranked R&D countries invested, on average, just 19 cents in R&D for each $100 of GDP in 2013—or about $9 per person.

### R&D landscape in retrospect (1980–2013)

**Fig 2:**
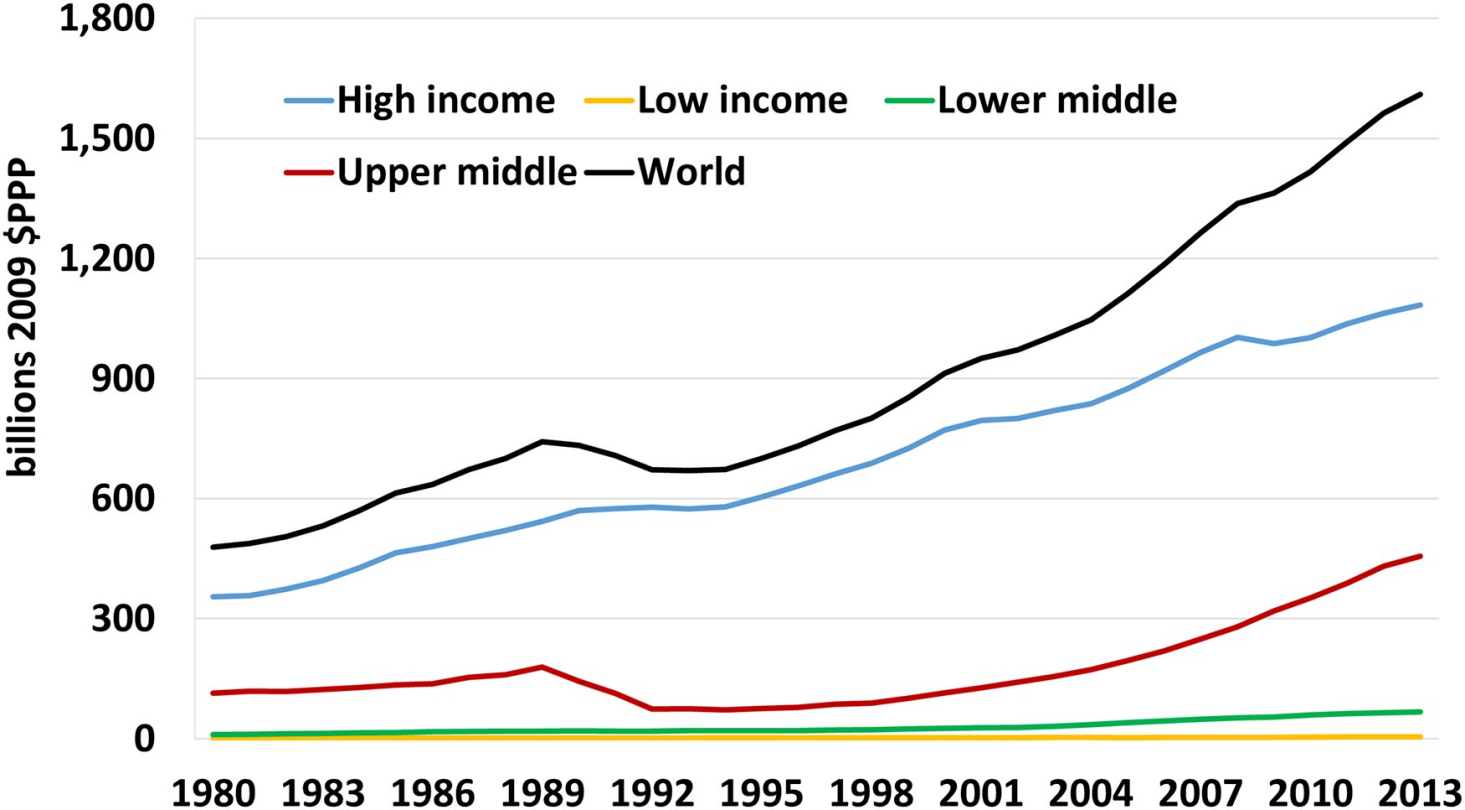
GERD spending by income group, 1980–2013. Source: Authors calculations. See S1 File for additional details. Note: The pronounced dip in the upper-middle income series in the early 1990s reflects the collapse in R&D spending that occurred with the dissolution of the Former Soviet Union.

The past three decades have seen substantial changes in the amount and patterns of R&D spending worldwide. Between 1980 and 2013, global investments in R&D increased from an estimated $478.6 billion (2009 PPP dollars) to $1.61 trillion (Fig 2 and Table 1). Global R&D spending grew at a slightly faster pace (3.52% per year in inflation-adjusted terms) than did economic output (GDP, 3.37% per year) such that the world’s economy became more research intensive. Spending $1.47 on R&D for every $100 of GDP in 1980 increased to $1.69 on R&D for every $100 of GDP in 2013. To put that growth in some perspective, in inflation-adjusted terms, U.S. spending on R&D in 2013 was almost equivalent to the entire world’s spending (including the United States) in 1980.

The pattern of growth in R&D spending varied markedly among countries. Starting from a relatively large base ($149.5 million 2009 PPP dollars in 1980), the rate of growth of U.S. investments in R&D was outpaced by the rest of the world, such that the U.S. share of global GERD shrank from 31.2% in 1980 to 27.4% in 2013. The Soviet Union was a major driving force in 1980, ranking second in overall R&D spending to the U.S., but by 2013, the 15 former Soviet states jointly accounted for just over half of their 1980 spending total when adjusted for inflation. Offsetting the declining scientific influence of Eastern Europe has been the ascendancy of several East and South Asian nations, notably China, Korea, and India. Remarkably, China’s global share alone grew from 1.2% to 19.2% over this period, jumping from 10^th^ to 2^nd^ ranked. Korea, now the world’s 5^th^ ranked R&D spender, ranked 36^th^ in 1980—also a remarkable climb in just 33 years. At the same time, India’s spending grew by an average of 6.7% per year, rising to 7^th^ from 13^th^ overall. Also notably, Brazil edged its way into the top 10 by increasing annual R&D expenditures by almost $29 billion since 1980, when it ranked 11^th^ in the world.

Growth in R&D spending can be parsed into the share of that change associated with overall economic growth (i.e., changes in GDP) and the share associated with the intensification of research spending (i.e., changes in R&D spending relative to GDP) using an indexing decomposition method developed by [27] (see also [13]). Over the period 1980–2013, about 88.6% of the growth in global GERD was attributable to country-level increases in GDP, such that as economies expanded (or contracted) so too was their GERD investment likely to increase (decrease) (Figure A2 in S1 File). Most distinctly, the overall research intensity in sub-Saharan Africa contracted over the period from 46 cents per $100 of GDP in 1980 to just 40 cents in 2013, meaning the entirety of the region’s modest growth in GERD was attributable to increases in the size of the region’s economy (which grew at 3.9% per year over this period).

#### Public vs. private research

Not only has the amount of R&D spending grown considerably over the past quarter of a century, the public versus private mix in the performance of that research has also changed markedly. Worldwide, the private share of GERD has increased over time, from 57.9% in 1980 to 66.0% in 2013 (Table 2). However, this structural shift towards R&D performed by private firms has occurred unevenly throughout the world. In 2013, 68.5% of high-income country R&D was performed by private firms, a comparatively small increase on the 64.6% share in 1980. The most dramatic change was among the upper-middle-income countries, whose private share grew from 40.7% in 1980 to 65.5% in 2013—just short of the corresponding high-income country share. The private share of research performed in lower-middle and low income counties is also on the rise, but research in these regions of the world is still primarily performed by public (i.e., government and university) institutions.

**Table 2:** Public vs private R&D, 1980 and 2013.

	GERD, 1980				GERD, 2013			
	Public	Private	Total	Private Share	Public	Private	Total	Private Share
	*(billion 2009 PPP$)*			%	*(billion 2009 PPP$)*			%
High Income	125.3	228.7	354.1	64.6	341.1	742.2	1,083.3	68.5
Upper Middle Income	67.2	46.2	113.3	40.7	157.2	298.3	455.5	65.5
Lower Middle Income	7.6	2.2	9.9	22.8	45.0	21.8	66.8	32.7
Low Income	1.1	0.2	1.3	13.2	3.5	0.8	4.4	19.3
**World**	**201**	**277**	**479**	**57.9**	**547**	**1,063**	**1,610**	**66.0**

Source: Authors calculations. See S1 File for additional details.

**Fig 3:**
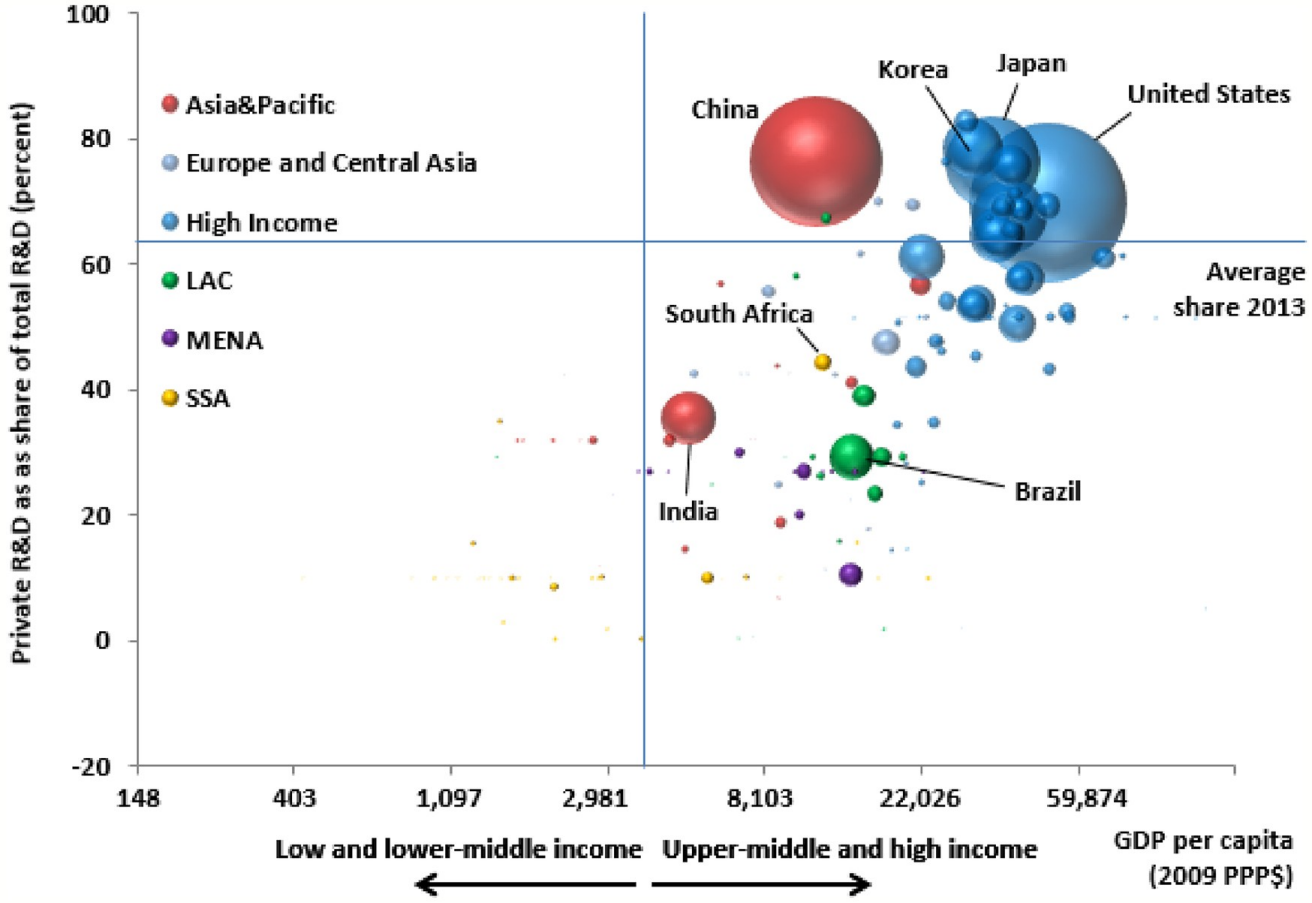
Private share of GERD by per capita GDP, 2013. Source: Authors calculations. See S1 File for additional details. Notes: GDP per capita plotted in natural logs. Size of bubble indicates country share of global GERD. Color of bubble indicates regional classification using World Bank schema [25]. Private R&D spending in China includes spending by state-owned, for-profit enterprises along with publicly listed private firms.

Fig 3 illustrates a positive association between the private share of R&D and per capita income such that the private sector share tends to be higher for more developed countries (i.e., with higher GDP per capita). The country bubbles in Fig 3 are scaled by the relative size of each country’s GERD, and color coded according to the region of the world in which the country is located. The rightward inclined orientation of the larger bubbles illustrates that the overall size of a country’s commitment to GERD is generally greater with higher GDP per capita and private sector R&D spending share.

### Prospective R&D landscapes (2014–2050)

Over the past three decades, even though changes in GDP accounted for most of the observed growth in GERD, many (but by no means all) countries also increased their intensity of R&D spending. Using the dynamic, country-specific, regression-based research intensities described in S1 File in conjunction with our compilation of alternative GDP forecasts, we projected a midline GERD scenario and two variants around that midline from 2014 to 2050. Looking ahead, the prospective changes in the global R&D landscape are dramatic. Allowing R&D intensities to change over time, the midline projection of spending on R&D puts the global total at $6.8 trillion (2009 PPP dollars) by mid-century (Table 1, and S3 Table). Alternatively, holding each country’s GERD intensity constant at its 2013 value, the projected midline global GERD total in 2050 would be $5.02 trillion (Table A7 in S1 File).

According to our (preferred) midline projections, the U.S. will command a shrinking share of the worldwide R&D total, down to around 14.4% by 2050. In contrast, this scenario has China spending 28.9% of the world’s R&D by 2050, double the corresponding U.S. total. If this seems implausible, note that this projected change in U.S.-China spending relativities is much more muted than the observed past change in R&D spending relativities between these two countries. In 1980, the U.S. spent 27.2-fold more than China, which had shrunk to just 1.4-fold more by 2013. Moreover, this midline projection has China increasing its inflation-adjusted spending about 6-fold from 2013 to 2050, which is comparatively modest growth relative to China’s 56-fold increase in real annual R&D spending from 1980 to 2013. In addition, China’s projected $1,412 of GERD per person in 2050 is still substantially lower than the 2050 GERD per-capita projection for the United States ($2,436 of GERD per person) (S4 Table).

Our midline projections have today’s middle-income countries (including China, India, and Brazil, among others) commanding the largest share of the global R&D landscape by the middle of the century, with 59.6% of the world’s R&D compared with a predicted share for today’s rich-countries of 39.7% (S3 Table). While the low-income country share more than doubles from 2013 to 2050, it is from a small base, such that this group of countries is projected to still spend a tiny share—just $49.3 billion (0.7%) of the projected $6.8 trillion 2050 global total.

**Fig 4:**
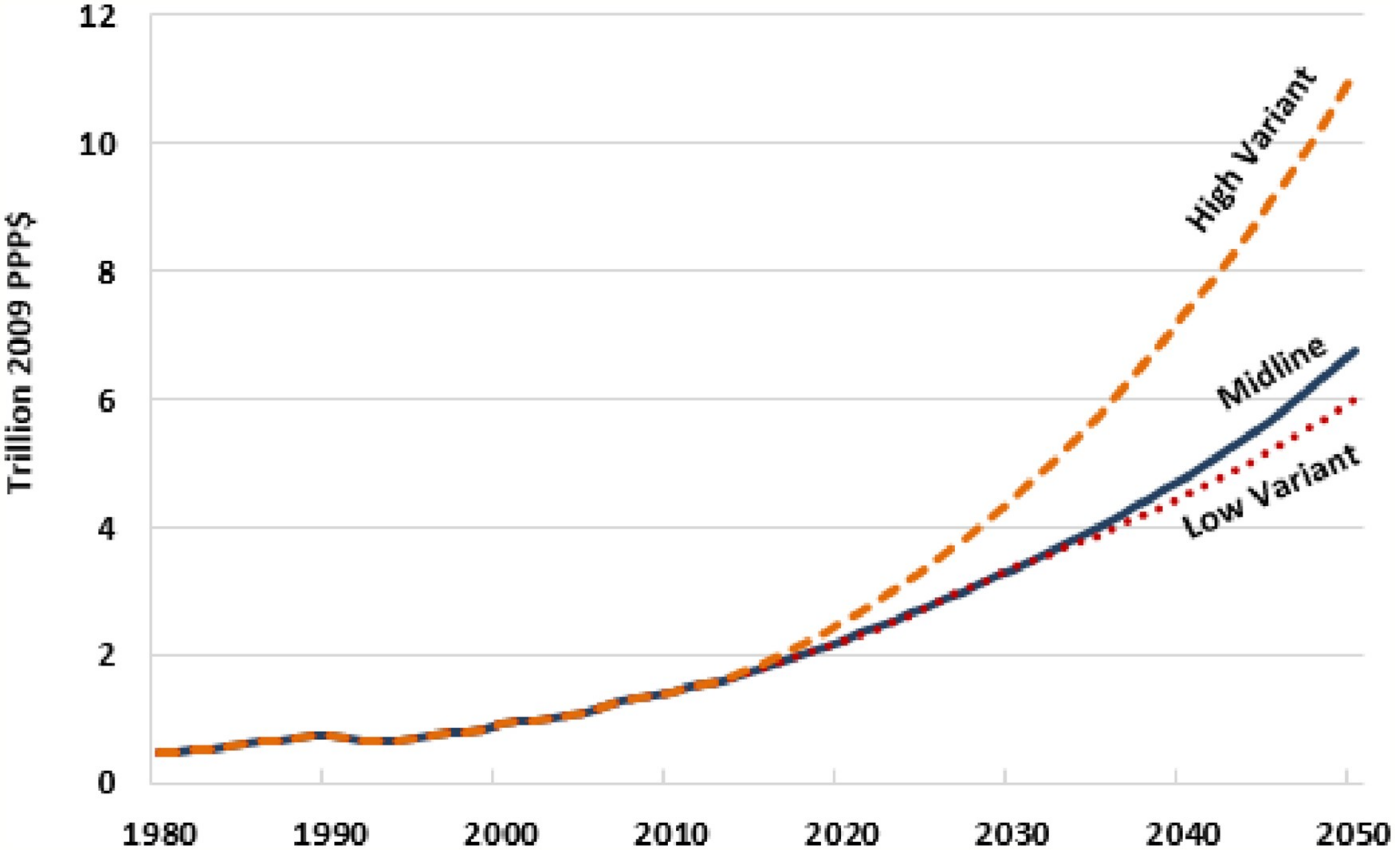
Historical GERD (1980–2013) and alternative projections (2014–2050). Source: Authors calculations. See S1 File for additional details.

The low and high projection variants imply that global GERD in 2050 could range from $6.02 trillion to as much as $11.13 trillion (Fig 4, dotted and dashed lines, respectively). This fairly wide range of prospective global research futures largely reflects the reported variation in the underlying mainstream GDP projections we sourced for this study (which deviate from the midline more substantially on the high end than the low end).

## Re-dealing the global R&D deck of cards?

The global R&D reshuffle over the past three decades has been largely limited to a small number of countries at the top. For example, in 1980, the U.S., Soviet Union, Japan, Germany, and France accounted for 72.3% of global R&D spending. By 2013, this group’s share (with the Former Soviet Republics exchanged for the Soviet Union) dropped to 48.6%, and by 2050. We project their share will fall further, to 26.6%, compared with a rise to a 47.8% share for Brazil, China, India, and Korea as a group. Of course, if Brazil’s recent and dramatic cuts in science spending (40% in the past three years) persist [28], this will reduce but by no means entirely undermine the size of the projected rise in this four-country global share during the decades ahead. These past and prospective geopolitical shifts in scientific standing among the leading nations are deserving of attention, given their influence in defining the global economic and political landscapes.

Still, the world remains dramatically divided between the scientific haves and the scientific have-nots: in 1980, the bottom 100 R&D ranked countries spent just 56 cents on R&D for every $100 spent by the top 10 ranked countries; by 2013, that figure had dropped to 51 cents for every $100. Our midline projections indicate that the scientific have-nots will continue losing ground relative to the rest of the world over the decades ahead, averaging only 38 cents for every $100 spent by the scientific haves in 2050. Perhaps most striking, 61 of the world’s 79 countries whose annual GERD was less than $15 per person in 1980, were still so in 2013—a small fraction of the $333 per person average for the top 10 ranked countries that same year.

The past and plausible future trends presented here point to a profound and economically important, albeit only partial, reshuffling of the global R&D deck. Spending on science does not necessarily equate to innovation, but as [29] describe, to the extent living standards are inextricably tied to the pace of productivity growth enabled by investments in R&D, these evolving global patterns of R&D are central to morbidity, mortality and quality-of-life outcomes. Our GERD projections indicate that left to their own R&D fates, the long-run economic growth prospects for many of the world’s poorest countries continues to look bleak. Indeed, the magnitude and persistence of the past and prospective gulf between the world’s scientific haves and have-nots calls for urgent policy and practical attention. History teaches us that one important institutional innovation would be to double down on enhancing the geographical spillovers of the benefits flowing from public and private R&D investments in richer countries if we are not to continue condemning large parts of the planet’s population to unacceptably low standards of living for a large part of the 21^st^ century.

## Supporting information

S1 Table Global gross expenditures on R&D, 1980–2013. (PDF)

S2 Table Global gross expenditures on R&D as a share of GDP and population, 1980–2013. (PDF)

S3 Table Projected global gross expenditures on R&D, 2015–2050. (PDF)

S4 Table Projected global gross expenditures on R&D as a share of GDP and population, 2015–2050. (PDF)

S5 Table Global growth in gross expenditures on R&D, 1980–2050. (PDF)

S1 File Supplementary material. (PDF)

S2 File Dataset. (XLSX)
