# Supplementary material for: Reshuffling the global R&D deck, 1980-2050
Source: PLoS One. 2019 Mar 29;14(3):e0213801. doi: 10.1371/journal.pone.0213801 (PMC6440631; doi:10.1371/journal.pone.0213801)
Supplement: S1 Table — (PDF) [file pone.0213801.s001.pdf]

S1 Table. Global gross expenditures on R&D, 1980-2013

	GERD								Share of World/Regional Total							
	1980	1985	1990	1995	2000	2005	2010	2013	1980	1985	1990	1995	2000	2005	2010	2013
	(Billions of 2009 international dollars)								*(Percent)*							
**High Income**	**354.1**	**464.0**	**569.5**	**603.7**	**771.0**	**874.2**	**1,002.3**	**1,083.3**	**74.0**	**75.6**	**77.7**	**86.2**	**84.4**	**78.6**	**70.8**	**67.3**
United States	149.5	201.0	227.8	243.9	329.0	356.7	404.4	441.2	42.2	43.3	40.0	40.4	42.7	40.8	40.3	40.7
Japan	49.3	72.6	100.5	105.1	118.8	139.0	138.9	153.4	13.9	15.6	17.7	17.4	15.4	15.9	13.9	14.2
Germany	42.9	50.9	59.9	54.1	67.8	70.8	84.5	94.0	12.1	11.0	10.5	9.0	8.8	8.1	8.4	8.7
Republic of Korea	1.1	4.3	9.3	15.6	20.8	31.4	50.6	62.3	0.3	0.9	1.6	2.6	2.7	3.6	5.0	5.8
France	24.2	29.8	37.3	39.0	42.4	45.0	49.4	51.0	6.8	6.4	6.5	6.5	5.5	5.1	4.9	4.7
United Kingdom	24.0	24.9	27.9	28.0	33.0	36.1	38.2	36.9	6.8	5.4	4.9	4.6	4.3	4.1	3.8	3.4
**Upper Middle Income**	**113.3**	**133.9**	**142.9**	**75.0**	**114.1**	**195.2**	**351.4**	**455.5**	**23.7**	**21.8**	**19.5**	**10.7**	**12.5**	**17.6**	**24.8**	**28.3**
China	5.5	8.3	11.8	16.3	41.5	95.6	217.8	308.8	4.9	6.2	8.3	21.7	36.4	49.0	62.0	67.8
Former Soviet Union	80.1	95.2	97.6	21.4	23.7	33.0	39.4	42.8	70.7	71.1	68.3	28.6	20.7	16.9	11.2	9.4
Brazil	5.5	5.0	11.2	14.2	19.0	21.0	31.1	34.1	4.8	3.7	7.9	18.9	16.6	10.8	8.9	7.5
Turkey	1.9	2.6	1.6	2.1	3.8	5.8	9.6	12.5	1.7	2.0	1.1	2.8	3.3	3.0	2.7	2.7
Iran	1.4	1.8	1.8	2.6	3.6	6.5	8.6	8.2	1.2	1.3	1.3	3.5	3.2	3.3	2.4	1.8
**Lower Middle Income**	**9.9**	**14.8**	**18.9**	**19.9**	**25.8**	**40.0**	**58.8**	**66.8**	**2.1**	**2.4**	**2.6**	**2.8**	**2.8**	**3.6**	**4.2**	**4.2**
India	4.8	9.0	12.2	13.3	19.3	29.4	43.4	50.6	49.0	60.7	64.5	66.6	74.8	73.6	73.7	75.7
Egypt	0.3	0.4	0.6	0.8	1.0	1.4	3.2	3.7	2.9	3.0	3.2	4.3	3.7	3.6	5.4	5.5
Pakistan	1.3	2.0	2.2	1.1	0.6	2.6	2.7	2.6	13.7	13.5	11.9	5.6	2.4	6.6	4.6	4.0
**Low Income**	**1.3**	**1.4**	**1.6**	**1.8**	**2.2**	**2.4**	**3.8**	**4.4**	**0.3**	**0.2**	**0.2**	**0.3**	**0.2**	**0.2**	**0.3**	**0.3**
Kenya	0.2	0.2	0.3	0.4	0.4	0.3	0.8	0.9	16.1	17.4	20.3	23.9	17.9	11.5	21.1	20.9
Tanzania	0.1	0.1	0.1	0.1	0.1	0.2	0.3	0.4	5.7	5.8	5.9	6.5	6.5	8.5	8.9	9.4
Uganda	0.05	0.1	0.1	0.1	0.1	0.1	0.3	0.3	3.6	3.8	4.2	5.8	4.5	6.0	7.0	7.1
Ethiopia PDR	0.05	0.05	0.1	0.1	0.1	0.2	0.2	0.3	3.7	3.4	5.3	5.0	5.8	7.0	6.2	7.2
**East/South Asia and Pacific**	**14.8**	**22.9**	**30.7**	**36.4**	**67.8**	**138.2**	**277.2**	**376.7**	**3.1**	**3.7**	**4.2**	**5.2**	**7.4**	**12.4**	**19.6**	**23.4**
**Europe and Central Asia**	**88.0**	**104.6**	**107.0**	**28.2**	**31.9**	**44.7**	**56.2**	**63.4**	**18.4**	**17.0**	**14.6**	**4.0**	**3.5**	**4.0**	**4.0**	**3.9**
**LAC**	**13.8**	**13.4**	**16.4**	**21.9**	**29.3**	**35.1**	**53.3**	**57.8**	**2.9**	**2.2**	**2.2**	**3.1**	**3.2**	**3.2**	**3.8**	**3.6**
**MENA**	**3.2**	**4.0**	**4.0**	**5.2**	**7.1**	**11.4**	**16.7**	**17.0**	**0.7**	**0.7**	**0.5**	**0.7**	**0.8**	**1.0**	**1.2**	**1.1**
**SSA**	**4.8**	**5.1**	**5.4**	**4.8**	**6.0**	**8.3**	**10.7**	**11.9**	**1.0**	**0.8**	**0.7**	**0.7**	**0.7**	**0.7**	**0.8**	**0.7**
**World Total**	**478.6**	**614.0**	**732.9**	**700.4**	**913.1**	**1,111.9**	**1,416.4**	**1,610.0**	**100.0**	**100.0**	**100.0**	**100.0**	**100.0**	**100.0**	**100.0**	**100.0**

Source: Authors calculations. See S1 File for additional details.

Notes: Bold entries are regional and global GERD totals and shares, other entries are country GERD totals and shares. These estimates are for 175 countries and country groups (with the 15 countries that were previously part of the former Soviet Union defined with reference to 1990 political geography). Countries grouped into income classes using 2013 World Bank schema (available at https://openknowledge.worldbank.org/handle/10986/21634). High-income countries are those with 2013 GNI per capita of $12,746 or more; upper middle-income countries had 2013 GNI per capita between $4,126 and $12,745; lower middle-income countries had 2013 GNI per capita between $1,046 and $4,125; and low-income countries had 2013 GNI per capita less than or equal to $1,045. Regional groupings are also based on the World Bank's classification of countries by region (available at http://data.worldbank.org/about/country-and-lending-groups). Countries of South Asia, East Asia and the Pacific are grouped into "East/South Asia and Pacific." "Europe and Central Asia" includes the Former Soviet Union, Turkey and the low- and middle-income countries of Eastern Europe. Country-specific shares are shares of respective income class.
