# Supplementary material for: Reshuffling the global R&D deck, 1980-2050
Source: PLoS One. 2019 Mar 29;14(3):e0213801. doi: 10.1371/journal.pone.0213801 (PMC6440631; doi:10.1371/journal.pone.0213801)
Supplement: S2 Table — (PDF) [file pone.0213801.s002.pdf]

S2 Table. Global gross expenditures on R&D as a share of GDP and relative to population, 1980-2013

	GERD as % of GDP								GERD per capita							
	1980	1985	1990	1995	2000	2005	2010	2013	1980	1985	1990	1995	2000	2005	2010	2013
	(GERD as a % of GDP)								*(2009 International Dollars per Person)*							
**High Income**	**1.8**	**2.0**	**2.1**	**2.0**	**2.1**	**2.2**	**2.3**	**2.4**	**390.2**	**491.8**	**581.9**	**594.9**	**735.2**	**803.2**	**886.2**	**940.3**
United States	2.3	2.7	2.5	2.4	2.6	2.5	2.7	2.8	649.4	831.2	895.1	909.8	1,156.2	1,196.5	1,295.1	1,378.6
Japan	2.2	2.6	2.8	2.8	3.0	3.3	3.3	3.5	425.0	605.0	822.3	844.0	944.8	1,094.8	1,090.6	1,206.1
Germany	2.4	2.6	2.6	2.1	2.4	2.5	2.8	2.9	542.3	644.7	744.0	650.7	811.6	844.4	1,018.3	1,136.8
Republic of Korea	0.6	1.5	1.9	2.2	2.3	2.7	3.7	4.2	29.3	106.3	215.3	348.9	452.4	667.5	1,043.4	1,264.5
France	1.9	2.1	2.3	2.2	2.1	2.1	2.2	2.2	449.2	538.4	655.5	672.9	715.4	732.3	781.7	793.5
United Kingdom	2.3	2.1	2.0	1.8	1.8	1.7	1.7	1.6	426.1	441.2	487.2	482.0	560.0	599.2	615.2	585.1
**Upper Middle Income**	**1.2**	**1.2**	**1.1**	**0.6**	**0.7**	**0.9**	**1.2**	**1.3**	**61.0**	**66.3**	**64.8**	**32.0**	**46.7**	**77.0**	**133.4**	**168.9**
China	0.8	0.7	0.7	0.5	0.9	1.3	1.8	2.0	5.6	7.8	10.2	13.2	32.4	72.5	160.2	222.9
Former Soviet Union	2.6	2.6	2.5	0.9	0.9	1.0	0.9	0.9	302.4	343.7	338.1	73.6	82.1	115.9	137.0	148.3
Brazil	0.4	0.4	0.8	0.8	1.0	1.0	1.2	1.2	44.8	36.7	75.1	87.5	108.8	113.1	159.5	170.1
Turkey	0.6	0.6	0.3	0.3	0.5	0.6	0.8	0.9	44.2	53.2	28.9	36.2	59.4	85.3	133.5	167.1
Iran	0.4	0.4	0.4	0.5	0.5	0.7	0.7	0.7	36.1	38.0	32.4	43.3	54.8	92.8	115.0	105.3
**Lower Middle Income**	**0.4**	**0.5**	**0.5**	**0.4**	**0.4**	**0.5**	**0.5**	**0.5**	**7.6**	**10.0**	**11.5**	**10.9**	**12.9**	**18.4**	**24.9**	**27.1**
India	0.6	0.8	0.8	0.7	0.7	0.8	0.8	0.8	6.9	11.5	14.0	13.9	18.5	26.1	36.0	40.4
Egypt	0.2	0.2	0.2	0.2	0.2	0.2	0.4	0.4	6.3	8.9	10.7	13.9	14.4	20.0	41.1	44.7
Pakistan	0.8	0.8	0.7	0.3	0.1	0.4	0.4	0.3	16.8	21.1	20.2	8.9	4.2	16.6	15.7	14.5
**Low Income**	**0.4**	**0.3**	**0.3**	**0.3**	**0.4**	**0.3**	**0.3**	**0.3**	**3.6**	**3.3**	**3.5**	**3.3**	**3.6**	**3.5**	**5.0**	**5.4**
Kenya	0.7	0.7	0.7	0.9	0.7	0.4	1.0	1.0	13.1	12.2	14.2	15.6	12.6	7.6	19.6	20.7
Tanzania	0.4	0.4	0.4	0.4	0.4	0.4	0.5	0.5	4.0	3.6	3.8	3.9	4.2	5.2	7.5	8.4
Uganda	0.6	0.6	0.6	0.6	0.4	0.4	0.6	0.6	3.8	3.6	4.0	5.0	4.1	5.0	7.8	8.3
Ethiopia PDR	0.2	0.2	0.3	0.2	0.3	0.3	0.2	0.2	1.3	1.1	1.7	1.5	1.8	2.1	2.5	3.1
**East/South Asia and Pacific**	**0.5**	**0.6**	**0.6**	**0.5**	**0.6**	**0.9**	**1.2**	**1.3**	**6.6**	**9.2**	**11.2**	**12.2**	**21.2**	**40.8**	**77.7**	**102.3**
**Europe and Central Asia**	**2.2**	**2.2**	**2.1**	**0.8**	**0.8**	**0.8**	**0.9**	**0.9**	**234.3**	**265.2**	**259.5**	**67.6**	**76.3**	**106.7**	**132.4**	**148.2**
**LAC**	**0.4**	**0.4**	**0.4**	**0.5**	**0.6**	**0.6**	**0.8**	**0.7**	**40.0**	**35.1**	**38.7**	**47.5**	**58.5**	**65.5**	**93.7**	**98.3**
**MENA**	**0.3**	**0.3**	**0.3**	**0.3**	**0.3**	**0.4**	**0.5**	**0.5**	**18.6**	**20.4**	**17.6**	**20.8**	**25.8**	**37.9**	**50.8**	**49.0**
**SSA**	**0.5**	**0.5**	**0.5**	**0.4**	**0.4**	**0.4**	**0.4**	**0.4**	**12.4**	**11.5**	**10.6**	**8.3**	**9.0**	**10.9**	**12.4**	**12.7**
**World Total**	**1.5**	**1.6**	**1.7**	**1.4**	**1.5**	**1.5**	**1.6**	**1.7**	**108.1**	**126.8**	**138.4**	**122.5**	**149.7**	**171.4**	**205.7**	**225.7**

Source: See Table 1.

Notes: See Table A1 in S1 File. Country-specific shares are shares of respective income class.
