# Supplementary material for: Reshuffling the global R&D deck, 1980-2050
Source: PLoS One. 2019 Mar 29;14(3):e0213801. doi: 10.1371/journal.pone.0213801 (PMC6440631; doi:10.1371/journal.pone.0213801)
Supplement: S3 Table — (PDF) [file pone.0213801.s003.pdf]

S3 Table. Projected global gross expenditures on R&D, 2015-2050

	GERD								Share of World/Regional Total							
	2015	2020	2025	2030	2035	2040	2045	2050	2015	2020	2025	2030	2035	2040	2045	2050
	(billions of 2009 international dollars)								*(Percent)*							
**High Income**	**1,166.0**	**1,388.3**	**1,612.9**	**1,832.0**	**2,048.0**	**2,256.2**	**2,465.2**	**2,680.0**	**66.0**	**62.3**	**58.5**	**54.8**	**51.2**	**47.4**	**43.5**	**39.7**
United States	481.8	570.0	641.4	705.0	768.5	834.2	902.9	976.5	41.3	41.1	39.8	38.5	37.5	37.0	36.6	36.4
Japan	157.5	171.5	190.1	206.0	219.0	229.0	238.4	248.1	13.5	12.4	11.8	11.2	10.7	10.2	9.7	9.3
Germany	99.2	110.4	116.8	120.2	123.6	128.3	134.1	141.0	8.5	8.0	7.2	6.6	6.0	5.7	5.4	5.3
Republic of Korea	62.9	72.1	85.4	101.5	120.0	135.8	149.7	160.3	5.4	5.2	5.3	5.5	5.9	6.0	6.1	6.0
France	56.5	71.8	86.2	98.2	108.1	116.9	126.0	135.7	4.8	5.2	5.3	5.4	5.3	5.2	5.1	5.1
United Kingdom	41.7	56.8	75.9	96.0	113.5	127.9	140.1	150.7	3.6	4.1	4.7	5.2	5.5	5.7	5.7	5.6
**Upper Middle Income**	**521.8**	**730.4**	**994.9**	**1,303.6**	**1,658.9**	**2,073.2**	**2,554.2**	**3,103.0**	**29.5**	**32.8**	**36.1**	**39.0**	**41.4**	**43.5**	**45.1**	**45.9**
China	358.9	515.9	708.3	915.5	1,131.7	1,367.9	1,638.2	1,955.3	68.8	70.6	71.2	70.2	68.2	66.0	64.1	63.0
Former Soviet Union	46.4	57.6	73.0	94.8	126.2	170.8	229.7	296.4	4.0	4.1	4.5	5.2	6.2	7.6	9.3	11.1
Brazil	38.5	53.8	75.9	107.0	146.9	190.0	230.2	266.6	7.4	7.4	7.6	8.2	8.9	9.2	9.0	8.6
Turkey	14.4	20.0	28.3	40.6	59.1	86.0	122.1	163.7	2.8	2.7	2.8	3.1	3.6	4.1	4.8	5.3
Iran	9.3	12.6	16.6	22.1	29.5	39.6	53.4	72.5	1.8	1.7	1.7	1.7	1.8	1.9	2.1	2.3
**Lower Middle Income**	**75.1**	**101.5**	**139.1**	**194.4**	**277.8**	**407.0**	**610.5**	**925.9**	**4.2**	**4.6**	**5.0**	**5.8**	**6.9**	**8.5**	**10.8**	**13.7**
India	57.4	79.5	111.9	160.6	236.0	355.4	547.2	848.3	76.4	78.3	80.4	82.6	84.9	87.3	89.6	91.6
Egypt	4.1	5.5	7.2	9.3	12.0	15.2	18.7	22.6	5.5	5.4	5.2	4.8	4.3	3.7	3.1	2.4
Pakistan	2.9	3.5	4.3	5.3	6.3	7.4	8.5	9.7	3.8	3.5	3.1	2.7	2.3	1.8	1.4	1.0
**Low Income**	**4.9**	**6.7**	**9.2**	**12.7**	**17.9**	**25.4**	**35.8**	**49.3**	**0.3**	**0.3**	**0.3**	**0.4**	**0.4**	**0.5**	**0.6**	**0.7**
Kenya	1.1	1.6	2.5	3.9	6.4	10.2	15.6	22.5	21.7	24.0	27.1	31.0	35.4	40.0	43.6	45.5
Tanzania	0.5	0.7	1.1	1.7	2.5	3.7	5.5	7.8	9.8	11.0	12.2	13.2	14.1	14.7	15.2	15.9
Uganda	0.4	0.5	0.8	1.1	1.6	2.3	3.4	4.8	7.3	7.8	8.3	8.7	9.0	9.2	9.4	9.8
Ethiopia PDR	0.4	0.5	0.6	0.8	0.9	1.1	1.2	1.3	7.4	7.3	6.9	6.1	5.2	4.2	3.4	2.7
**East/South Asia and Pacific**	**435.3**	**619.9**	**852.3**	**1,119.2**	**1,426.2**	**1,802.3**	**2,288.2**	**2,931.0**	**24.6**	**27.8**	**30.9**	**33.5**	**35.6**	**37.8**	**40.4**	**43.4**
**Europe and Central Asia**	**69.9**	**90.3**	**119.1**	**160.5**	**220.1**	**304.1**	**413.5**	**537.6**	**4.0**	**4.1**	**4.3**	**4.8**	**5.5**	**6.4**	**7.3**	**8.0**
**LAC**	**64.3**	**85.7**	**115.5**	**156.5**	**208.5**	**265.4**	**319.8**	**371.1**	**3.6**	**3.8**	**4.2**	**4.7**	**5.2**	**5.6**	**5.6**	**5.5**
**MENA**	**19.2**	**25.6**	**33.8**	**45.0**	**60.0**	**80.0**	**106.0**	**139.8**	**1.1**	**1.1**	**1.2**	**1.3**	**1.5**	**1.7**	**1.9**	**2.1**
**SSA**	**13.2**	**17.1**	**22.3**	**29.6**	**39.7**	**53.8**	**73.1**	**98.7**	**0.7**	**0.8**	**0.8**	**0.9**	**1.0**	**1.1**	**1.3**	**1.5**
**World Total**	**1,767.9**	**2,226.8**	**2,756.1**	**3,342.8**	**4,002.6**	**4,761.7**	**5,665.7**	**6,758.3**	**100**	**100**	**100**	**100**	**100**	**100**	**100**	**100**

Source: Baseline projections. See S1 File (including Table A1) for additional details.

Notes: Country-specific shares are shares of respective income class.
