# Supplementary material for: Reshuffling the global R&D deck, 1980-2050
Source: PLoS One. 2019 Mar 29;14(3):e0213801. doi: 10.1371/journal.pone.0213801 (PMC6440631; doi:10.1371/journal.pone.0213801)
Supplement: S4 Table — (PDF) [file pone.0213801.s004.pdf]

S4 Table. Projected global gross expenditures on R&D as a share of GDP and relative to population, 2015-2050

	GERD as % of GDP								GERD per capita							
	2015	2020	2025	2030	2035	2040	2045	2050	2015	2020	2025	2030	2035	2040	2045	2050
	(GERD as a % of GDP)								*(2009 International Dollars per Person)*							
**High Income**	**2.5**	**2.7**	**2.8**	**2.9**	**3.0**	**3.1**	**3.1**	**3.1**	**1,001.3**	**1,163.7**	**1,325.2**	**1,480.7**	**1,633.8**	**1,781.5**	**1,930.5**	**2,084.7**
United States	3.0	3.2	3.4	3.4	3.4	3.5	3.5	3.5	1,481.9	1,686.4	1,829.4	1,944.1	2,057.7	2,177.2	2,302.7	2,436.1
Japan	3.5	3.5	3.5	3.5	3.5	3.5	3.5	3.5	1,242.3	1,368.2	1,541.9	1,707.7	1,861.6	1,999.8	2,141.1	2,290.1
Germany	3.1	3.3	3.4	3.4	3.4	3.5	3.5	3.5	1,201.6	1,348.1	1,444.8	1,511.3	1,584.0	1,680.7	1,799.5	1,943.6
Republic of Korea	3.8	3.6	3.5	3.5	3.5	3.5	3.5	3.5	1,263.5	1,420.0	1,655.7	1,944.3	2,288.6	2,598.5	2,891.8	3,140.5
France	2.4	2.8	3.2	3.3	3.4	3.4	3.4	3.5	868.8	1,078.2	1,268.7	1,416.9	1,533.0	1,634.5	1,740.5	1,853.2
United Kingdom	1.8	2.1	2.6	3.0	3.2	3.4	3.4	3.4	652.8	866.5	1,128.8	1,398.2	1,624.5	1,801.2	1,942.8	2,060.1
**Upper Middle Income**	**1.4**	**1.6**	**1.9**	**2.1**	**2.3**	**2.4**	**2.5**	**2.6**	**190.8**	**259.3**	**345.6**	**446.2**	**562.4**	**699.5**	**861.5**	**1,051.2**
China	2.2	2.6	3.0	3.3	3.4	3.4	3.4	3.5	256.1	360.0	488.8	629.9	781.2	952.9	1,158.5	1,411.8
Former Soviet Union	1.0	1.1	1.2	1.4	1.7	2.1	2.5	2.9	160.6	198.9	253.3	332.2	447.2	611.6	831.3	1,086.9
Brazil	1.3	1.5	1.8	2.2	2.7	3.1	3.3	3.4	189.2	254.8	349.0	480.3	647.9	828.3	997.1	1,153.6
Turkey	1.0	1.1	1.3	1.5	1.8	2.2	2.7	3.1	187.3	249.1	337.8	467.5	660.0	937.4	1,306.0	1,730.6
Iran	0.8	0.8	0.9	1.0	1.1	1.3	1.5	1.8	117.3	149.5	189.0	241.7	312.8	409.0	539.8	720.6
**Lower Middle Income**	**0.5**	**0.5**	**0.6**	**0.6**	**0.7**	**0.8**	**0.9**	**1.1**	**29.6**	**37.2**	**47.9**	**63.3**	**85.9**	**120.3**	**173.2**	**253.5**
India	0.8	0.9	1.0	1.1	1.3	1.6	1.9	2.3	44.8	58.7	78.8	108.8	154.7	227.0	342.7	523.6
Egypt	0.4	0.4	0.4	0.4	0.4	0.4	0.4	0.3	48.8	60.0	73.9	91.1	111.5	134.4	159.1	185.8
Pakistan	0.3	0.3	0.3	0.3	0.3	0.2	0.2	0.2	15.2	17.3	19.8	22.7	25.8	29.0	32.4	35.6
**Low Income**	**0.3**	**0.3**	**0.3**	**0.3**	**0.4**	**0.4**	**0.4**	**0.5**	**5.8**	**7.0**	**8.7**	**11.0**	**14.1**	**18.3**	**23.7**	**30.3**
Kenya	1.0	1.2	1.3	1.6	1.9	2.4	2.8	3.1	22.9	30.4	41.9	59.6	86.3	124.9	175.0	231.1
Tanzania	0.5	0.5	0.5	0.5	0.5	0.5	0.5	0.6	9.3	12.1	16.1	21.3	28.0	36.4	47.1	60.6
Uganda	0.6	0.6	0.6	0.6	0.6	0.6	0.7	0.7	9.0	11.1	13.9	17.5	22.2	28.3	36.2	46.5
Ethiopia PDR	0.2	0.2	0.2	0.2	0.2	0.2	0.1	0.1	3.4	4.1	4.8	5.3	5.8	6.1	6.4	6.6
**East/South Asia and Pacific**	**1.4**	**1.6**	**1.8**	**2.0**	**2.1**	**2.1**	**2.2**	**2.3**	**115.9**	**158.0**	**209.8**	**267.8**	**333.9**	**415.5**	**522.6**	**666.7**
**Europe and Central Asia**	**0.9**	**1.1**	**1.2**	**1.4**	**1.7**	**2.1**	**2.5**	**2.8**	**162.8**	**208.7**	**274.7**	**371.1**	**511.3**	**710.4**	**973.1**	**1,277.7**
**LAC**	**0.8**	**0.9**	**1.0**	**1.1**	**1.3**	**1.4**	**1.5**	**1.5**	**107.0**	**135.6**	**175.0**	**228.3**	**294.9**	**366.0**	**432.7**	**495.2**
**MENA**	**0.5**	**0.5**	**0.5**	**0.6**	**0.6**	**0.7**	**0.7**	**0.8**	**53.6**	**66.1**	**81.7**	**102.6**	**130.2**	**165.9**	**211.3**	**269.5**
**SSA**	**0.4**	**0.4**	**0.4**	**0.4**	**0.4**	**0.4**	**0.4**	**0.5**	**13.4**	**15.2**	**17.7**	**20.9**	**25.0**	**30.4**	**37.4**	**46.0**
**World Total**	**1.7**	**1.9**	**2.0**	**2.1**	**2.1**	**2.2**	**2.2**	**2.3**	**242.3**	**289.7**	**342.3**	**398.3**	**459.5**	**528.7**	**610.8**	**710.1**

Source: Baseline projections. See S1 File (including Table A1) for additional details.

Notes: Country-specific shares are shares of respective income class.
