# Supplementary material for: Reshuffling the global R&D deck, 1980-2050
Source: PLoS One. 2019 Mar 29;14(3):e0213801. doi: 10.1371/journal.pone.0213801 (PMC6440631; doi:10.1371/journal.pone.0213801)
Supplement: S5 Table — (PDF) [file pone.0213801.s005.pdf]

S5 Table. Global growth in gross expenditures on R&D, 1980-2050

	1980-2013 by decade and overall					
	**1980-1990**	**1990-2000**	**2000-2010**	**2010-2013**	**1980-2013**	**2013-2050**
	(Percent per year)					
**High Income**	**5.26**	**3.08**	**3.02**	**2.61**	**3.43**	**2.42**
United States	4.80	3.70	2.62	3.07	3.30	2.00
Japan	7.49	1.78	2.08	3.11	3.18	1.34
Germany	3.94	1.27	2.32	3.51	2.17	0.91
Republic of Korea	25.34	9.24	9.40	6.84	11.32	2.90
France	4.33	1.02	1.34	1.07	2.15	2.48
United Kingdom	2.06	1.92	1.91	-1.33	1.74	3.86
**Upper Middle Income**	**3.91**	**-0.59**	**12.09**	**9.23**	**3.49**	**5.26**
China	8.27	12.68	18.18	12.60	13.65	4.94
Former Soviet Union	4.23	-11.06	5.09	2.99	-3.47	5.55
Brazil	8.74	5.31	5.51	2.84	6.42	6.01
Turkey	-1.85	6.65	11.07	9.16	5.79	7.40
Iran	1.70	6.53	9.61	-1.64	6.43	5.99
**Lower Middle Income**	**7.25**	**3.10**	**9.48**	**4.24**	**5.50**	**7.35**
India	10.18	4.85	9.51	5.06	6.65	7.93
Egypt	8.14	4.40	9.79	4.32	7.40	5.09
Pakistan	6.96	-14.79	19.12	-0.47	1.07	3.63
**Low Income**	**2.66**	**2.79**	**4.22**	**4.98**	**3.49**	**6.87**
Kenya	4.46	0.60	6.65	4.55	3.28	9.37
Tanzania	2.65	3.97	8.60	6.80	5.33	8.37
Uganda	3.42	4.14	9.03	5.32	5.53	7.73
Ethiopia PDR	7.59	7.55	2.25	9.70	5.72	3.88
**East/South Asia and Pacific**	**7.94**	**7.89**	**15.54**	**10.95**	**10.19**	**5.52**
**Europe and Central Asia**	**4.03**	**-9.46**	**5.89**	**4.24**	**-2.51**	**6.15**
**LAC**	**1.57**	**5.97**	**6.84**	**2.76**	**4.97**	**5.41**
**MENA**	**2.14**	**5.56**	**8.32**	**0.65**	**5.78**	**5.86**
**SSA**	**0.64**	**0.91**	**6.23**	**3.68**	**2.92**	**5.93**
**World Total**	**5.01**	**2.51**	**4.82**	**4.40**	**3.52**	**3.88**

Source: Baseline R&D estimates for the period 1980-2013, and midline projections thereafter.

Note: All growth rates were adjusted for inflation and were calculated using the least-squares method described in [31, p.409]. Europe and Central Asia include 9 countries from 1980 to 1989 (Albania, Bulgaria, Bosnia and Herzegovina, Hungary, Macedonia, Romania, Turkey, Serbia, and Montenegro), 10 countries in 1990 and 1991 (the nine countries listed previously and Kosovo), and 21 countries after 1991 (the previously listed countries and Armenia, Azerbaijan, Belarus, Georgia, Kazakhstan, Kyrgyzstan, Republic of Moldova, Tajikistan, Turkmenistan, Ukraine, Uzbekistan).
