# Supplementary material for: Reshuffling the global R&D deck, 1980-2050
Source: PLoS One. 2019 Mar 29;14(3):e0213801. doi: 10.1371/journal.pone.0213801 (PMC6440631; doi:10.1371/journal.pone.0213801)
Supplement: S1 File — (PDF) [file pone.0213801.s006.pdf]

Supplementary Material

February 26, 2019

* This supplementary material includes documentation for the construction of InSTePP's GERD version 3.5 series plus additional GERD projections details in support of the paper by S.P. Dehmer, P.G. Pardey, J.M. Beddow and Y. Chai. "Reshuffling the Global R&D Deck, 1980-2050."

OUTLINE

1. Constructing Global Estimates of Gross (Public and Business) Domestic Expenditure on R&D (GERD, PERD and BERD), 1980-2013 1

1.1 Estimating GERD Intensities 2

GERD (Intensities) in China 3

GERD (Intensities) in the Former Soviet Republics 3

1.2 Estimating GERD 4

Estimating Public (PERD) and Business (BERD) R&D Spending 4

Consistency with other InSTePP Innovation Accounts 4

2. Projecting the Global Gross Domestic Expenditure on R&D (GERD) Series, 2014-2050 5

2.1 GERD Projections Methodology 6

Drivers of GERD Changes 6

2.2 Projecting GERD Intensities 9

Examining Past and Prospective GERD Intensities 10

2.3 Projecting GDP 12

Examining Past and Prospective GDP 14

2.4 GERD Projections: Description and Diagnostics 17

References 19

Figures and Tables 23

1. Constructing Global Estimates of Gross (Public and Business) Domestic Expenditure on R&D (GERD, PERD and BERD), 1980-2013[1]

The National Science Foundation reports country-specific GERD (gross domestic expenditure on research and development) estimates for 61 (many high-income) countries for circa 2013 (NSB 2016, Table 4-4) and a 93-country total for each of the years 2013, 2008 and 2005. To develop these estimates NSF drew on their own data and that available via the OECD (Organization for Economic Cooperation and Development) and UNESCO (United Nations Educational, Scientific and Cultural Organization (NSB 2016, p. 4-36). Our objectives in constructing this InSTePP series were to:

- extend and refine the country GERD coverage, paying particular attention to developing estimates for lower-income countries (or regions) to supplement available estimates for the generally richer countries, and
- extend the InSTePP GERD series back in time, well before the presently available NSF series that begins in 1996, to a series that spans the period 1980 to 2013.

R&D trends, and the effects of R&D, take time to reveal themselves. A comprehensive series stretching back over three decades expands analytical possibilities and our ability to render policy-relevant conclusions.

The series was constructed in several, interlinked steps. First we formed a series of annual, country-specific GERD intensities—defined as the quotient of total R&D spending to gross domestic product (GDP) for each country-year for the period 1980-2013. The GERD intensities, in conjunction with GDP estimates taken mainly from the United Nations (2014a), were used to form gross expenditure on research and development (GERD) estimates in 2009 international (i.e., purchasing power parity, PPP) dollars. There are 6,554 country-years in this global R&D series, with direct estimates—as distinct from interpolated or econometrically derived estimates—for 1,763 (27.2 percent) of these country-years, which constitute 93.7 percent of the overall $30.5 trillion of global R&D spending (2009 international dollars) for the period 1980-2013. A number of replicable methods to back-cast, forward-cast and interpolate missing GERD intensities were developed and implemented as described in detail below. We also developed estimates of the shares of public versus private R&D to parse the GERD totals into their respective public and private research components.

[1] The authors thank Connie Chan-Kang for her outstanding assistance in the preparation of this report.

1.1 Estimating GERD Intensities

The core annual, country-specific GERD intensities for the years 1980-2013 were compiled from five primary sources: Eurostat (2014), OECD (2015), RICYT (2015), UNESCO (2015) and the World Bank (2015). We considered all sources to be equally reliable and authoritative, and therefore derived our GERD intensities using the mean of the reported intensities across all the core sources from which we could obtain data. This methodological decision was inconsequential as the standard deviations of research intensities across these primary sources are very small. We also identified GERD intensity estimates that were not included in the online UNESCO database from a number of older published UNESCO sources (2005, 1999, 1998, 1996, and 1993). When estimates could not be derived from any of the primary sources identified above, we filled gaps in the data by using mean intensities taken from these published UNESCO sources. The source data files, their dates of creation and access, and the number of countries and the time period included in each series are summarized in Table A1.

[Table A1: *Primary Data Sources for GERD Intensity Estimates*]

Three approaches were used to extrapolate data for a number of country-years from the core set of research-intensity data described above: within-country interpolation, within-country extrapolation and between-country extrapolation.

Within-country interpolation: gaps in research intensities for any given country, defined as having known values at times T_a and T_b, but missing values between these times, were interpolated assuming a constant growth rate between the values at times T_a and T_b. Specifically, the constant growth rates were approximated using the following formula:

$$r = \left(\frac{Value_{T_b}}{Value_{T_a}}\right)^{\frac{1}{T_b - T_a}} - 1,$$

where r is the constant growth rate, $Value_{T_a}$ is the reported value at time T_a and $Value_{T_b}$ is the reported value at time T_b (*Ta* < *Tb*).

Within-country extrapolation: missing research intensity values at the beginning or end of a time series for any given country were obtained by extrapolating backward or forward a constant value from the earliest or latest known value (i.e., assuming a constant intensity ratio, when no better information is available), respectively.

Between-country extrapolation: for countries with no GERD research-intensity data, estimates were constructed by calculating the weighted average research intensity for peer economies in each country's

geographical region (see country classifications by region in Table A2). Brazil, China, India, Nigeria and South Africa were excluded from these calculations because their science and technology economies are distinctive relative to their geographic neighbors. For example, our estimate of the average research intensity in Latin America and the Caribbean for 1990 is 0.43 percent with Brazil included, and 0.21 percent with Brazil excluded.

[Table A2: *Regional Classifications for InSTePP Global GERD Database*]

GERD (Intensities) in China

The growth in Chinese GERD is a notable feature of these data, and so special attention was devoted to forming these estimates. Secondary estimates of Chinese GERD were drawn from the following sources: UNESCO (1996 and 1998) for 1981; UNESCO (1999) for the period 1988-1999; OECD (2015) for 1990-1995; OECD (2015), UNESCO (2015) and World Bank (2015) for 1996-2000, and; Eurostat (2015), OECD (2015), UNESCO (2015) and World Bank (2015) for 2001-2012. Our resulting Chinese GERD estimates track very closely with those reported by Sun and Cao (2014, Table A2). For example, using our GERD intensities and corresponding GDP estimates, our year-2000 estimate (in current local currency units) is 90.8 billion versus 89.57 billion reported by Sun and Cao (2014), and our 2011 figure is 881.6 billion compared with Sun and Cao's estimate of 868.7 billion.

GERD (Intensities) in the Former Soviet Republics[2]

This version of the InSTePP GERD series reaches back to an era that pre-dates the collapse of the Soviet Union, a period when Soviet investments in science were substantial. Tracking the volatile changes in Soviet science spending since 1980 is thus critical to measuring and assessing overall changes in global GERD over this period, and, like the Chinese data, were also given special attention. The foundation of our GERD estimates for the Former Soviet Republics comes from R&D expenditures reported in the official Union of Soviet Socialist Republics (USSR) statistical volumes (GOSKOMSTAT, Central Statistical Bureau of the USSR). We used the average adjustment for the years 1987-1989 estimated by Gokhberg and Midely (1993) to account for an acknowledged double-counting between agencies (among other issues) for the years 1980-1990, yielding an adjusted R&D expenditure total that is, on average, about 72 percent of the officially reported figure.

[2] We thank Leonid Gokhberg for his insightful guidance (personal correspondence) that helped in forming these EE&FSU GERD estimates, and also thank Helen Trenz for her excellent assistance in translating various source documents.

The official USSR statistical volumes report three measures of gross economic output: gross social product (GSP), gross national product (GNP), and national income produced. According to at least one (now declassified) report by the U.S. Central Intelligence Agency (1996), gross social product is a "heavily double-counted statistic summing the outputs of all sectors of the economy without netting out the intermediate sales from one producing sector to another." Unfortunately, this is the most widely reported figure across the statistical volumes. The more useful measure, gross national product (which is deemed consistent with United Nations accounting principles) is only reported in the later volumes. This left missing GNP values for 1981-1984. For 1981, 1982, and 1984, we used the mean ratio of GSP/GNP for all available years over the period 1980-1990 to impute GNP. We did not find a statistical volume that reported GSP or GNP for 1983, so we set this value at the midpoint of the 1982 and 1984 values.

Combining the adjusted GERD and GNP estimates, we calculated a GERD:GNP ratio for the USSR for the years 1980 to 1990. R&D spending was not reported in the official statistical volumes for 1991, so for this year, we applied a 30 percent decline in research intensity estimated for Russia between 1990 and 1991 in line with Gokhberg et al. (1997).

1.2 Estimating GERD

With a complete panel of GERD intensity ratio estimates in hand, we estimated GERD (in 2009 international (i.e., PPP) dollars) by multiplying these country-specific research intensities by the corresponding GDP time-series expressed in 2009 international dollars taken from UN (2013).

Estimating Public (PERD) and Business (BERD) R&D Spending

To parse the GERD totals into their respective public (i.e., public expenditure on R&D, PERD) and private (i.e., business expenditure on R&D, BERD) components we used the "by performer" data from the GERD intensity sources identified in Table A1. Public shares were calculated as unity less the share performed by business or industry (i.e., the for-profit sector). For countries without reported public share data, estimates were constructed by calculating the weighted average public share for any given country's geographical region (see country classifications by region in Table A2). Again, when forming these regional averages, data from the high-income countries along with Brazil, China, India, Nigeria and South Africa were excluded to remove their (distinctive) influences on the respective regional averages.

Consistency with other InSTePP Innovation Accounts

As a final consistency check, the GERD estimates were compared with the corresponding InSTePP version 3.5 public food and agricultural R&D estimates whose construction is described in Pardey et al.

(2015). Our rationale for this adjudication exploited differences in the source data used to generate estimates for each of the series. Namely, the data and sources used to generate the food and agricultural R&D estimates for many small and low-income countries in particular were more attainable and deemed more reliable compared with those used to derive the corresponding GERD estimates. Moreover, the economies of many low- (and lower-middle-) income countries are much more heavily reliant on agriculture, and so one would expect their R&D portfolios to be also so inclined. Therefore, if our GERD estimate was considered abnormally low relative to the corresponding agricultural R&D estimate, we adjusted the GERD estimate *ex post* with a carefully-considered, albeit *ad hoc* but fully documented, approach. Specifically, our initial GERD estimate was adjusted if the ratio of publicly performed agricultural R&D to publicly performed GERD was greater than 80 percent. In such instances, GERD was adjusted to equal the following: (non-ag GDP * GERD/GDP) + public ag R&D + (public ag R&D/public GERD ratio)*(1 - public GERD ratio).[3] In words, the estimated GERD research intensity is applied to GDP net of agriculture to estimate the non-agriculture portion of GERD. To this portion, publicly- and privately-performed agricultural R&D is added, with the private agricultural R&D component estimated by assuming that the private-to-public ratio for agricultural R&D is equivalent to the corresponding ratio for overall R&D within the country. This last step is done because we do not have direct estimates of private food and agricultural R&D for most countries requiring this adjustment for consistency.

2. Projecting the Global Gross Domestic Expenditure on R&D (GERD) Series, 2014-2050

To project global GERD we take advantage of the identity relationship that for any particular country in a given year, the product of that country's intensity of research (i.e., GERD/GDP ratio) and its corresponding GDP equals the country's gross expenditure on R&D for that particular year. On the assumption that the empirical regularities of the past will persist over the coming decades, we used our newly developed historical GERD series for the period 1980-2013, in conjunction with an ensemble of GDP forecasts obtained from various sources, to form projections of regional and global GERD to 2050.

[3] The countries for which GERD estimates were recalibrated based on cross tabulation with the public food and agricultural R&D estimates are: Angola, Bangladesh, Belize, Burkina Faso, Burundi, Cameroon, Cape Verde, Central African Republic, Colombia, Congo, Rep., Costa Rica, Cote d'Ivoire, Cyprus, Ecuador, El Salvador, Eritrea, Ethiopia, Fiji, Gambia, The, Ghana, Guatemala, Guinea, Guyana, Honduras, Indonesia, Jamaica, Kenya, Lao PDR, Lesotho, Liberia, Madagascar, Malawi, Malaysia, Mali, Mauritania, Mauritius, Morocco, Myanmar, Namibia, Nepal, Nicaragua, Niger, Nigeria, Pakistan, Papua New Guinea, Paraguay, Philippines, Puerto Rico, Qatar, Rwanda, Samoa, Senegal, Singapore, Solomon Islands, Sri Lanka, St. Lucia, Swaziland, Togo, Tuvalu, Uganda, Uruguay, Vanuatu, and Zambia. The consistency check condition was typically met and the adjustment applied for country-years when direct agricultural R&D estimates were available and the GERD estimates were obtained indirectly.

Section 2.1 lays out the empirical basis for the GERD projections methodology we developed and implemented. The GERD intensity and GDP futures we envisage are each developed and discussed in sections 2.2 and 2.3 respectively. Section 2.4 concludes this report with an assessment of the robustness of our midline GERD projections in light of plausible variation in the research intensity and GDP estimates that underpin them.

2.1 GERD Projections Methodology

As we demonstrate in the sub-section below, "Drivers of GERD Change," research intensity ratios (i.e., GERD/GDP) for a given country tend to exhibit more temporal stability than do the corresponding GERD time series. Thus, we proceed by projecting research intensity ratios, and then applying those ratios to GDP projections to derive GERD projections. This method was particularly appealing because research intensity ratios exhibit significant cross-sectional regularity, and could therefore be estimated without relying on time-series techniques. The practical implications for long-run projections are:

- The projections are based on a well-specified data generating process rather than a purely statistical approach. This means that out-of-sample projections are more likely to be valid, and that theory-based *a priori* signs and magnitudes can be specified for the estimated parameters. The out-of-sample validity is further enhanced by employing cross-sectional data rather than trend-based projections.
- For a given country-year, GERD is the product of the corresponding intensity ratio and GDP. Thus, the well-documented and generally robust GDP projections are fully and transparently utilized in deriving our GERD estimates.
- While this projections method has the distinct advantages of parsimony and replicability, it means that our global GERD projections are sensitive to variation in the estimates of prospective GDP.

Drivers of GERD Changes

For a given country-year, GERD exactly equals the product of the corresponding GERD intensity ratio and GDP. Thus, following Beddow and Pardey (2015), economic index number methods can be applied via this identity to shed light on the drivers of changes in research expenditures. Namely, Paasche- and Laspeyres-type intensity ratio indexes of GERD can be defined, respectively, as:

$$P_{IR} = 100\frac{\sum_i IR_{it} \cdot GDP_{it}}{\sum_i IR_{ib} \cdot GDP_{it}} \qquad (1)$$

and

$$L_{IR} = 100\frac{\sum_i IR_{it} \cdot GDP_{ib}}{\sum_i IR_{ib} \cdot GDP_{ib}} \quad (2)$$

where *t* is the (rolling) current year and *b* is the base year of the index series. The Paasche-type intensity ratio index reveals the current-year GERD index-value that would have occurred had all countries increased their GDP to current-year levels in the base year. The Laspeyres version gives the index value that would have occurred had GDP not changed.

Further, the corresponding GDP indexes of GERD are:

$$P_{GDP} = 100\frac{\sum_i IR_{it} \cdot GDP_{it}}{\sum_i IR_{it} \cdot GDP_{ib}} \quad (3)$$

and

$$L_{GDP} = 100\frac{\sum_i IR_{ib} \cdot GDP_{it}}{\sum_i IR_{ib} \cdot GDP_{ib}}. \quad (4)$$

Just as the product of a Paasche quantity index and a Laspeyres price index yields a simple index of total expenditure, the product of a Paasche intensity ratio index and the corresponding Laspeyres GDP index (or *vice versa*) exactly equals a simple index of GERD (see Beddow and Pardey (2015) for a proof of this in the context of crop output). This relationship can be exploited to generate counterfactual scenarios that shed light on the sources of GERD changes. Here, using a base year of 1980, the 2013 index values are:

$P_{IR} = 139$, $L_{IR} = 118$, $P_{GDP} = 284$, and $L_{GDP} = 242$

Thus,

$$\frac{P_{IR} \cdot L_{GDP}}{100} \equiv \frac{L_{IR} \cdot P_{GDP}}{100} = 336$$

The (relevant) interpretation is that changes in GDP increased GERD by 2.42 to 2.84 times, holding each country's intensity ratio at its 1980 and 2013 level, respectively. Correspondingly, the changes in each country's intensity ratio increased GERD by 1.18 and 1.39 times, holding each country's GDP at its 1980 and 2013 level, respectively. Together, the changes in GDP and intensity ratio increased GERD by 3.36 times. [4]

Figure A1 reports the results of using this indexing method to decompose the sources of GERD growth for countries grouped into various income classes (Figure A1 Panel a) and geographic regions

[4] Appendix Table A1 summarizes the rates of growth in GERD, GERD intensities, and GDP for the period 1980-2013 for key countries and countries grouped into high-, middle- and low-income classes. Unless otherwise stated, all growth rates in the text and tables are calculated using the least squares regression method, where the rate is revealed as the slope coefficient of a regression of logarithms of variables against trend, as described in World Bank (2011, p. 409).

(Figure A1 Panel b). All of the GDP indexes (upper part of Figure A1 Panel a) increased over time, indicating that GDP growth accounts for some of the corresponding growth in GERD over time, and especially for the lower-middle income countries whose Paasche and Laspeyers GDP indexes increased the fastest. For the most part, the respective intensity ratios also increased over time (Figure A1 Panel b), indicating that the intensification of R&D spending has also contributed to the growth in GERD. However, a comparison of Panels a and b in Figure A1 indicates that the rate of growth in GDP generally outpaced the rate of intensification of R&D, such that over time a larger share of GERD growth is attributable to growth in the size of the economy rather than the intensification of R&D spending. Some notable discrepancies in these general trends are a) the precipitous and marked drop in the intensity of research of the upper-middle income countries beginning in 1990 (owing to the collapse of the former Soviet Union), and b) the decline in research intensity of the low-income group of countries during the 1980 to 2013 period (meaning that all of their GERD growth during this period is attributable to an increase in GDP). When grouped by geographic regions (Figure A1 Panel b), the Asia & Pacific group of countries had the highest rate of growth in GDP growth since 1980, while the Europe and Central Asian group of countries experienced either stagnant or declining growth in GDP. For the Europe and Central Asian group of countries, their regional intensity ratio index (lower part of Figure A1 Panel b) collapsed during the late 1980s, once again a reflection of developments following the collapse of the former Soviet Union.

[Figure A1: *Indexed Decomposition of the Sources of GERD Growth, 1980-2013*]

The indexing approach described in equations (1) to (4) and reported in Figure A1 above captures the effect of changes in *both* the levels *and* the relative GERD shares across countries, whereas a standard logarithmic-difference decomposition captures the drivers of *aggregate* global changes in GERD. Using logarithmic differences, GDP growth accounted for 88.6 percent of the increase in global GERD over the 1980-2013 period, while growth in GERD intensity accounted for 11.4 percent of the increase (Figure A2). There are substantial differences in regional patterns of growth. Mirroring the developments described above, the research intensity of the low-income group of countries has shrunk since 1980, meaning the region's GERD growth is entirely attributable to its GDP growth. For the lower-middle-income and high-income countries, the average share of GERD growth attributable to growth in GDP is 76.7 and 71.4 percent, respectively, so that growth in each region's intensity ratio accounted for less than 30 percent of the overall GERD growth. For the upper-middle-income group of countries, the collapse of the former Soviet Union is once again evident in the income class (see upper-middle income image in Panel a, and Europe and central Asia image in Panel b) in Figure A2. All other regions (Asia &

Pacific, LAC, MENA, and High Income) exhibited positive growth in both GDP and intensity ratios throughout the study period.

[Figure A2: *Logarithmic Difference Decomposition of the Sources of GERD Growth, 1980-2013*]

2.2 Projecting GERD Intensities

The historical GERD and GDP data show that the recent (2003-2013) rate of growth in research intensity tends to increase at a decreasing rate with the level of research intensity (see Figure A3). Because knowledge stocks are generated via a cumulative process, the data imply that the marginal benefit of R&D investments at the country level might be lower for those countries that have especially low research intensities as they are less able to leverage historical investments. Further, the data reveal that countries begin to reach R&D satiation as intensity ratios increase above unity. To capture this relationship, we regressed the 2003-2013 growth rate in R&D intensity on the 2013 level of R&D intensity, specifying the independent variable as a second order polynomial to capture the decreasing marginal effect.[5] Specifically, we estimated the following equation:

$$G_i^{IR} = \beta_0 + \beta_1 IR_i + B_2 IR_i^2 + \epsilon \qquad (5)$$

where

G_i^{IR} is the 2003 to 2013 continuous growth rate in the research intensity ratio for country i, and

IR_i is the 2013 intensity ratio for country i.

[Figure A3: *GERD Intensity, 2013*]

The estimated parameters and the regression overall were significant at any reasonable level of statistical significance (i.e., $\rho < 0.01$). Next, the results were used to project the 2013 country intensity ratios through to 2050, taking account of country-level temporal dynamics, namely:

$$IR_{it} = (IR_{it-1})e^{G(IR_{it-1})} \qquad (6)$$

where

[5] We used a deliberate procedure to choose the period 2003-2013 as the basis for our country-level growth rates. First, we opted to use 2013 as the terminal year to exploit the most recent data available. Second, we did not consider years prior to 1993 because of the dramatic global geopolitical and structural shifts that occurred around that time. Further, the longer run intensity growth rate becomes unstable with initial years beginning after 2007, so we considered initial years ranging from 1993 to 2007. The dynamic model was run using growth rates derived with each prospective initial year in this range, all with a terminal year of 2013. None of these initial years yields a global GERD projection for 2050 that differed by more than 3.1 percent versus the chosen baseline, and most generated a substantially similar estimate. Using 2003 as the initial year yields a 2050 global GERD estimate that differs by only 0.16 percent from the median GERD estimated derived using each of the other candidate initial years.

IR_{it} is the predicted intensity ratio for country *i* in year t,

$G()$ is the estimated relationship between the growth rate in country intensities and the corresponding lag of the intensity level, and

e is the base of natural logarithms.

Examining Past and Prospective GERD Intensities

So, what does the extended history made possible by these new InSTePP GERD estimates reveal about the changing structure of GERD intensities worldwide, and how does that compare with the global prospects for GERD intensities that we foresee? Figure A4, Panel a plots the simple average of GERD intensities for countries grouped into three per capita income groups (high, medium and low) and the world; Panel b presents GERD intensities for the same country grouping but this time in terms of weighted averages for the period 1980-2013. Notably, while worldwide average GERD intensities increased modestly, from 1.47 in 1980 to 1.69 in 2013 (Panel b) this was not universally true. Average intensities for high-income countries grew from 1.76 in 1980 to 2.37 in 2013 whereas they decreased slightly for sub-Saharan African countries (from 0.46 in 1980 to 0.40 in 2013). Notably, the research intensity for the Former Soviet Republics decreased sharply from 2.58 in 1980 to 0.92 in 2013, pulling the middle-income average down with it.

[Figure A4 *GERD Intensity Trends, 1980-2050*]

Figure A4, Panel b shows a precipitous decline in GERD intensities for the middle-income countries from a local peak of 1.207 in 1989 down to 0.546 just three years later in 1992, owing to the rapid collapse of the Soviet Union's economy and with it spending on Soviet science.[6] In 1989, the Soviet Union constituted 68.6 percent of the middle-income GERD total, shrinking to just 28.1 percent of that total in 1992. The average GERD intensity for the high-income countries also dipped from 2.086 in 1991 to 1.951 in 1994 (Figure A4, Panel b), a reflection of the early 1990s recession that hit the United States and other high-income countries (Temin 1998).

[6] Countries were categorized into income groups according to their 2013 gross national income (GNI) per capita and the classification schema reported in World Bank (2015, p. xiii). As the data described here reflect a 1990 world map, GNI and population for countries that split after 1990 were re-aggregated. For example, the Former Soviet Union (FSU) aggregate was placed in the upper middle income group. Dividing the total 2013 GNI of the Former Soviet Republics by the corresponding 2013 total population revealed that the average GNI per capita for the FSU was US$9,140, which falls within the middle-income range according to World Bank's definition (whose groupings are: low income, $1,045 or less; lower middle income, $1,046–4,125; upper middle income, $4,126–12,745; and high income, $12,746 or more).

The box and whisker plots in Figure A5 are a more informative version of Figure A4, Panel a. Each panel not only plots the simple average GERD intensity for each income class and the world, but also indicates the country-specific dispersion in GERD intensities around the income group average for each year from 1980 to 2013. The top and bottom endpoints of the whisker represent the simple average GERD intensity for the top 5 and bottom 5 countries respectively; the bottom and top of the box are the first and third quartiles; and the segment where the dark and light green area meet represent the median. Clearly, irrespective of the income class, there is considerable variation in country-specific GERD intensities around the respective averages, with no apparent tendency for that dispersion to change in any systematic way over the past quarter of a century.

[Figure A5: *GERD Intensity Trends: Income Averages and Country Level Dispersions, 1980-2013*]

While the global (weighted) average GERD intensity increased from 1.47 in 1980 to 1.69 in 2013 (Figure A4, Panel b), 37 percent of the 175 countries in our GERD intensity sample reduced their GERD intensities over this period of time, and the GERD intensity increased slowly (by less than one percent per year) for 28 percent of the countries in the sample (Table A3).[7] About half of the low- and middle-income countries in both Latin America and the Caribbean and sub-Saharan Africa decreased their research intensities while about 69 percent of those countries in the Middle East & North Africa increased their research intensity. Notably, over three quarters of the high-income countries increased their research intensity even though those countries already had research intensity ratios above the global average in 1980.

[Table A3: *Average Rates of Growth of Country-Level GERD Intensities, 1980-2013*]

Table A4 provides an historical perspective on rates of growth of GERD plus the level and rates of growth of GERD intensities. With countries grouped into per capita income classes, the average GERD intensity for all three income classes either shrank or grew more slowly during the 1990s than for either the preceding or the following decades. GERD intensities grew most rapidly for the high-income countries during the 1980s (averaging 1.76 percent increase per year), and even though that growth slowed a little (averaging 1.56 percent per year) during the 2000s, GERD intensity growth in the high-income countries continued to outpace the corresponding GERD intensity growth in both the low- and

[7] The main data inputs include the 199 World Bank countries as described above. However, country fragmentation, especially after 1990, mean that the data inputs do not yield a balanced panel. We opted to take a "1990" world view to generate a consistent country coverage for our historical and projected GERD series. This results in a dataset with 175 country and country aggregates that has the advantage of not requiring additional assumptions to generate temporally consistent regional and country aggregates. Here and in the main paper, "country" refers to these 175 aggregates unless otherwise stated (or clear from the context).

middle-income countries during this decade. Notably over the entire 1980-2013 period the global average GERD intensity grew by just 0.11 percent per year, well below the average rate of growth in overall GERD which increased by 3.7 percent per year.

[Table A4: *Historical GERD Growth Rates and GERD Intensity Ratios and Growth Rates, 1980-2013*]

2.3 Projecting GDP

We used the model-generated GDP projections (2005 PPP dollars) produced by the Centre D'études Prospectives et D'informations Internationales (CEPII) (Fouré et al. 2012) to establish inflation-adjusted GDP growth rates for our base case scenario. When GDP forecasts were unavailable for a particular country, we projected that country's GDP forward by applying the mean projected growth rate, by year, for the country's region. For each of the respective regions, we excluded Brazil, China, India, Nigeria and South Africa from these calculations, both because we had direct estimates for each of them and because their GDP growth projections are generally distinctive relative to their geographic neighbors. To generate our low and high scenarios of GDP (and, correspondingly GERD), we extrapolated GDP using the minimum or maximum growth rate, respectively, reported by country and year across an additional eight sources of GDP projections (Dadush and Stancil, 2010; U.S. Department of Agriculture Economic Research Service 2012; Fogel 2007; Goldman Sachs 2007; Ward 2011; Maddison 2007; Organisation for Economic Co-operation and Development 2012; and Pricewaterhouse Coopers 2011). Details of the data coverage from each source are described below.

Carnegie (2010): This report contains GDP projections for 19 countries for 2009, 2030, and 2050 in constant (2005) US dollars (Page 8). Included countries were: Argentina, Australia, Brazil, Canada, China, France, Germany, India, Indonesia, Italy, Japan, Korea, Mexico, Russia, Saudi Arabia, South Africa, Turkey, the United Kingdom, and the United States. They also presented constant real annual GDP growth rates for the period 2009-2005, and these are the estimates we used in our calculations.

CEPII (2012): This report and companion database (Fouré et al. 2012) contains GDP projections for 147 countries for years 2010-2050 in constant (2005) US dollars. For years 2010-2012, projections are based on International Monetary Fund short-term forecasts, and for years 2013-2050, projections are based on the MaGE (Macroeconometrics of the Global Economy) model, which incorporates capital accumulation, the savings rate, the relationship between the savings and investment rate, education, female participation, and technological progress in its forecasts. We converted these projections into annual arithmetic growth rates for use in our calculations.

Fogel (2007): This NBER working paper contains individual country GDP growth rate projections for China, India, Japan and the United States and grouped GDP growth rate projections for 15 European Union countries, 6 Southeast Asian countries, and the "rest of the world" for the period 2000-2040 (Table 3, Page 6). The 15-country European group includes: Austria, Belgium, Denmark, Finland, France, Germany, Greece, Ireland, Italy, Luxembourg, Netherlands, Portugal, Spain, Sweden, and the United Kingdom; the 6-country Southeast Asian group includes: Singapore, Malaysia, Indonesia, Thailand, South Korea, and Taiwan. The presented average annual growth rates are constant over the full period and presumed to be in real terms (based on constant 2000 US dollars). For our calculations, we attributed the growth rates to all named countries, and all others were assumed to follow Fogel's "rest of world" projected growth rate.

Goldman Sachs (2007): This report contains GDP projections for 22 countries in 5-year increments from 2010-2050 in constant (2005) US dollars (Page 149). Included countries are: Bangladesh, Brazil, Canada, China, Egypt, France, Germany, India, Indonesia, Iran, Italy, Japan, South Korea, Mexico, Nigeria, Pakistan, Philippines, the Russian Federation, Turkey, the United Kingdom, the United States, and Vietnam. We converted these projections into annual arithmetic growth rates for use in our calculations.

HSBC (2011): This report contains GDP growth rate projections for 40 countries in 10-year increments from 2010-2050 (Table 30, page 22). The estimates are based on a Robert Barro growth model and appear to be inflation-adjusted. Included countries are: Argentina, Australia, Austria, Belgium, Brazil, Canada, China, Colombia, Denmark, Egypt, Finland, France, Germany, Greece, Hong Kong, India, Indonesia, Iran, Ireland, Israel, Italy, Japan, South Korea, Malaysia, Mexico, Netherlands, Norway, Poland, Russian Federation, Saudi Arabia, Singapore, South Africa, Spain, Sweden, Switzerland, Thailand, Turkey, United Kingdom, United States, and Venezuela.

Maddison (2007): This book contains GDP projections for 20 countries and 8 global regions from 2003-2030 in constant (1990) international dollars (Table 7.9, page 343). Implied annual real growth rates were calculated using the linear arithmetic growth formula. Included countries are: Australia, Brazil, Canada, China, France, Germany, India, Indonesia, Iran, Italy, Japan, South Korea, Mexico, Russian Federation, Spain, Taiwan, Thailand, Turkey, United Kingdom, and United States. Included regions are: Africa, Eastern Europe, Latin America, Other Asia, Other Former USSR, Other Western Offshoots, Western Europe, and Rest of World.

OECD (2012): This database contains GDP growth rate projections for 42 countries for the time intervals 1995-2011, 2011-2030, and 2030-2060 for real (2005=100 base) international dollars. Included countries are: Argentina, Australia, Austria, Belgium, Brazil, Canada, Chile, China, Czech Republic, Denmark, Estonia, Finland, France, Germany, Greece, Hungary, Iceland, India, Indonesia, Ireland, Israel, Italy, Japan, Luxembourg, Mexico, Netherlands, New Zealand, Norway, Poland, Portugal, Russian Federation, Saudi Arabia, Slovak Republic, Slovenia, South Africa, South Korea, Spain, Sweden, Switzerland, Turkey, United Kingdom, and United States.

PwC (2011): This report contains real GDP growth rate projections for 22 countries for the period 2009-2050 (Table 5, Page 20). Included countries are: Argentina, Australia, Brazil, Canada, China, France, Germany, India, Indonesia, Italy, Japan, Mexico, Nigeria, the Russian Federation, Saudi Arabia, South Africa, South Korea, Spain, Turkey, the United Kingdom, the United States, and Vietnam. The presented average annual growth rates are constant over the full period.

PwC (2015): This report was consulted for revised GDP growth projections of China, India, and the Former Soviet Republics based on findings reported by Pritchett and Summers (2014).

USDA, ERS (2012): This data set contains GDP projections for 188 countries for years 2010-2050 in constant (2005) US dollars. We converted these projections into annual arithmetic growth rates for use in our calculations.

Examining Past and Prospective GDP

The GERD futures we developed hinge heavily on the published empirical evidence we canvassed regarding global GDP prospects. The primary sources we used to construct the low, midline and high GDP projections for each of the year's 2015-2050 are given in Table A5. Most projections (65.8 percent) in our base case scenario were derived from country-specific estimates reported in CEPII (2012), with the remaining (25.4%) based on average regional rates. PwC 2015 was consulted in our base scenario to update GDP projections for China, India, and the Former Soviet Republics due to findings reported by Pritchett and Summers (2014) as described below. CEPII (2012) data also figured heavily in the 2015-2030 period for the high-end projections (53.8%) and constituted an even larger share of both the low- and high-end projections over the 2031-2050 period (60.0 percent and 71.0 percent, respectively), in part because this was the only published source available to us for 21.5 percent of the estimates during this latter period. Maddison (2007) was a significant source of the low-end GDP projections (51.7 percent of the estimates for the 2015-2030 period and 20.8 percent for the period 2031-2050), but no

other source accounted for more than 20 percent of our estimates for any of the periods or income scenarios summarized in Table A3.

[Table A5: *Sources for GDP Projections for Midline, Low and High Estimates, 2015-2050*]

Setting aside the PwC (2015) estimates (see below), the implied growth rates of the midline GDP estimates that underpin the GERD estimates are in Table A6. They show global GDP growing by 3.3 percent per year over the period 1980-2013, with a slightly lower rate of long-run growth being projected for the period 2013-2050 (3.1 percent per year). Notably, the growth of rich-country economies has been slowing over time; averaging 2.7 percent per year for the period 1980-2013, 1.8 percent per year for the past decade or so (specifically the period 2000-2013), and projected to grow at the rate of 1.7 percent per year for the period 2013-2015. During the 1980s and 1990s, Sub-Saharan Africa's GDP grew at a relatively slow pace (1.2 percent per year in the 1980s, but increased to 2.5 percent per year during the 1990s). The region's rate of economic growth accelerated to 5.7 percent per year during the 2000-2013 period, around the pace of growth projected through to 2050.

[Table A6*: Past and Projected Rates of GDP Growth, 1980-2050*]

China's GDP grew at a remarkably high and reasonably steady 10 percent per year for the 1980-2013 period, but in line with very recent developments and general expectations, the country's long-run rate of GDP growth is projected to slow.[8] As with all such long-term projections, just how much slower is open to speculation. Citing the regression-to-the mean evidence of Pritchett and Summers (2014), PwC (2015) report a projected growth rate of 3.5 percent per year through to 2050, which is slower than the projection of 4.9 percent per year using the CEPII (2012) estimates. We are persuaded by the Pritchett and Summers (2014) findings, and opted for the slower PwC projected long run rate of GDP growth for our midline estimate for China.[9] Likewise, projections from CEPII (2012) were for the Indian economy to

[8] It appears that this process has already begun. China's GDP grew at an annual rate of 14.2 percent per year in 2007, 10.6 percent per year in 2010, slowing to 7.3, 6.9, and 6.7 percent per year in years 2014, 2015 and 2016 respectively (World Bank 2017).

[9] Pritchett and Summers (2014, p. 2) discussed the prospects of a continuation of rapid (greater than 4 percent per year) growth rates for countries like China and India over the long run. They conclude that "Consensus forecasts for the global economy over the medium and long term predict the world's economic gravity will substantially shift towards Asia and especially towards the Asian Giants, China and India. While such forecasts may pan out, there are substantial reasons that China and India may grow much less rapidly than is currently anticipated. Most importantly, history teaches that abnormally rapid growth is rarely persistent, even though economic forecasts invariably extrapolate recent growth. Indeed, regression to the mean is the empirically most salient feature of economic growth." They go on to observe that (p. 37) "… the typical (median) end of an episode of super-rapid growth is near complete regression to the world mean growth rate. … deceleration of that magnitude would take India's current growth episode of 6.29 to 1.64 percent and China's from 8.63 (in the episode since 1991) to 3.98 percent."

grow at 5.3 percent per year through to 2050, whereas PwC (2015) report a projected growth of 4.9 percent per year. Similarly, the PwC projected growth for Russia was 2.1 percent per year (for the period 2014-2050), well below the CEPII (2012) view of 4.3 percent per year. Again for our midline projection, we opted for the more conservative PwC projection for both India and the Former Soviet Republics (using Russia as a proxy for the FSR growth rate).

Figure A6, Panel a plots the global GDP trend for the period 1980-2013 plus the high, midline and low projections of GDP though to 2050. Compared with the historical average growth of 3.4 percent per year (for the period 1980-2013), the projected midline rate of growth is 3.1 percent per year (for the period 2013-2050). The high projection has an average rate of growth of 4.8 percent per year, compared with 2.6 percent per year for the low projection. The terminal 2050 global GDP estimates are $295.5 trillion for the midline (2009 PPP dollars), $530.3 trillion for the high projection and $244.9 trillion for the low projection (compared with $95.5 trillion in 2013).

[Figure A6*: GDP and GDP Per Capita Projections]*

To further help assess the plausibility of these projections we used population data from UN (2013) to form a number of GDP per capita estimates (Figure A6, Panel b). Global per capita GDP grew by 1.9 percent per year from an average of $7,357 per person (2009 PPP dollars) in 1980 to $13,383 per person in 2013. The midline projection has global per capita income growing by 2.3 percent per year from 2013-2050 to an average of $31,045 per person in 2050. The high projection has global growth averaging 4.0 percent per year with a terminal value of $55,717 in 2050, compared with growth of just 1.8 percent per year for the low-end projection (terminal value of $25,734 per person).

The box and whiskers plot in Figure A6, Panel b gives an indication of the changing distribution of per capita income across the 199 countries in this series using the observed data from 1980-2013 and the midline projected estimates for the period 2013-2015. The top and bottom endpoints of the whisker represent the simple average of per capita GDP for the top 30 and bottom 30 countries respectively; the bottom and top of the box are the first and third quartiles; and the segment where the dark and light green areas meet represents the median. As in Panel a of this same figure, the (historical and projected midline, low, and high) trend lines of GDP per capita are weighted averages. A notable characteristic of these data is a gradual increase in the cross-country variation of per capita income over time, both within the historical series and (more pronounced) in the projected series. However, the spread in average per capita income between the top and bottom 30 countries declined from a factor of 50.0 in 1980—i.e., an average of $940 per person (2009 PPP dollars) for the bottom 30 countries versus $47,010 per person for the top 30 countries—, to 43.1 in 2013 (i.e., $1,332 versus $57,459 per person), and 22.3 in 2050 (i.e., $4,612 versus $102,070 per person). Average per capita incomes for the top 30 countries

grows by 1.3 percent per year over the entire 1980-2050 period compared with growth of 2.5 percent per year for the bottom 30 countries.

2.4 GERD Projections: Description and Diagnostics

Based on the identity $GERD_{it} = IR_{it} \cdot GDP_{it}$, GERD intensity projections (IR_{it}) for each country *i* were applied to various GDP projections to generate plausible bounds for our GERD projections. Table A7 summarizes details of our midline GERD projection and variants around the midline. Using the dynamic, country-specific, regression-based research intensities described above, we compute a midline scenario and two variants of that baseline. The midline GERD projection uses the midline GDP projection series as described above.

[Table A7: *GERD Projections Details—Midline and its Variants, with 2050 Endpoints*]

The midline scenario assumes China's GDP will grow by 3.5 percent per year from 2013-2050 (compared with 5.0 percent per year for the high variant). Had the CEPII (2012) based GDP projections for China been applied, global GERD would have been projected to grow by 4.5 percent per year (versus 3.9 percent per year for the midline) to total $8.19 trillion (versus $6.67 trillion) by 2050. Under this scenario, projected Chinese GERD would total $3.39 trillion in 2050 (compared with $1.96 trillion for the midline scenario), which is still substantially greater than the $977 billion midline GERD projected for the United States in 2050. Under the midline scenario, global GERD intensity is projected to be 2.29 percent in 2050 versus 2.43 percent had the CEPII (2012) GDP projections been applied

The low and high GERD variants imply that global GERD in 2050 could range from $6.02 trillion to as much as $11.13 trillion. This fairly wide range of prospective global research futures results exactly because of the inherent difficulty of projecting GDP. It is worth noting that the 2050 global weighted average intensity ratio is outside the bounds of what has been observed historically, but the underlying country intensity ratios generate GERD series that are well in-line with the corresponding country trends.

To get a handle on the potential importance of our projected shift in intensity ratios, we explored two additional scenarios in which intensity ratios were held constant. For the first additional scenario, we held each country's GERD intensity constant at its 2013 value and applied the midline GDP projections to estimate global GERD through to 2050. This procedure yielded a 2050 global GERD value of $4.02 trillion (2009 PPP dollars). However, while this bounds our estimates to the historically observed intensity ratios, it generates implausible country-level trends. For example, this forces China's

GERD intensity to suddenly stop growing whereas the historical data show China's intensity ratio to be growing at rapid pace (5.9 percent per year) over the latest decade in the data (2003-2013).

The second additional scenario held the *global* GERD intensity constant at its 2013 value and applied this intensity to midline global GDP estimate to project global GERD to 2050. The resulting global GERD projections were then allocated to each country in proportion to its current-year share of global GDP, yielding a 2050 global GERD value of $4.98 trillion.[10]

[10] We also explored other alternatives, such as holding the global intensity ratio constant and allocating global GERD to countries such that they maintained their 2013 share of global GERD. However, we found little compelling theoretical or intuitive support for such static alternatives given the profound shifts in global GERD spending reported here.

References

Text

Beddow, J.M. and P.G. Pardey. “Moving Matters: The Effect of Location on Crop Production.” *The Journal of Economic History* 75, 1 (March 2015) 219-249.

Central Intelligence Agency. "CIA Meets the Press: Soviet Economic Slowdown and CIA Make Headlines." CIA Historical Review Program. Volume 13, No. 2. 1996. Downloaded January 1, 2013 from www.cia.gov/library/center-for-the-study-of-intelligence/kent-csi/vol13no2/html/v13i2a02p_0001.htm.

National Science Board. *Science and Engineering Indicators 2014*. Arlington VA: National Science Foundation, 2014a.

National Science Board. *Science and Engineering Indicators: 2014 Digest*. Arlington VA: National Science Foundation, 2014b.

Pardey, P.G., C. Chan-Kang, S.P. Dehmer and J.M. Beddow. *DOCUMENTATION: InSTePP’s International Innovation Accounts-Research and Development Spending Series, version 3.5*. St Paul: University of Minnesota, International Science and Technology Practice and Policy Center, 2015.

PwC (PricewaterhouseCoopers). *The World in 2050: Will the Shift in Global Economic Power Continue*. United Kingdom: PricewaterhouseCoopers, 2015. Downloaded July 22, 2015 from www.pwc.com/gx/en/issues/the-economy/assets/world-in-2050-february-2015.pdf.

Pritchett, L. and L.H. Summers. “Asiaphoria Meets Regression to the Mean.” NBER Working Paper 20573. Washington, D.C.: National Bureau of Economic Research, October 2014. Available at http://www.nber.org/papers/w20573.

Sun, Y and C. Cong. “Demystifying Central Government R&D Spending in China: Supplementary Material.” *Science* 345, 1006 (August 2014).

Temin, P. "The Causes of American Business Cycles: An Essay in Economic Historiography," in J.C. Fuhrer and S. Schuh, eds., *Beyond Shocks: What Causes Business Cycles?* Federal Reserve Bank of Boston, 1998.

United Nations Statistics Division. *UN National Accounts Main Aggregates Database*. New York: United Nations (2013). Downloaded September 2nd 2014 from http://unstats.un.org/unsd/snaama/introduction.asp. Last update December 2013.

United Nations. “National Accounts Main Aggregates Database.” New York: United Nations, 2013a. Accessed on September 2nd, 2014 from: http://unstats.un.org/unsd/snaama/dnlList.asp. Last update: December 2013.

United Nations, Department of Economic and Social Affairs, Population Division. “World Population Prospects: The 2012 Revision”. New York: United Nations, 2013b. Accessed on October 29th 2013 from: http://esa.un.org/wpp/index.htm. Last update: June 2013.

World Bank. ‘’GDP Growth (annual %).” Washington, D.C: World Bank, 2017. Available at http://data.worldbank.org/indicator/NY.GDP.MKTP.KD.ZG?locations=CN.

World Bank. ‘’The World by Income’’ in *World Development Indicators 2015*. Washington, D.C: World Bank, 2015.

World Bank. *World Development Report 2012: Gender Equality and Development*. Washington, D.C.: World Bank, 2011.

Data Sources

Central Intelligence Agency. "CIA Meets the Press: Soviet Economic Slowdown and CIA Make Headlines." CIA Historical Review Program. Volume 13, No. 2. 1996. Downloaded January 1, 2013 from www.cia.gov/library/center-for-the-study-of-intelligence/kent-csi/vol13no2/html/v13i2a02p_0001.htm.

Dadush, U. and B. Stancil. *The World Order in 2050*. Washington, DC: Carnegie Endowment for International Peace, 2010. <http://carnegieendowment.org/files/World_Order_in_2050.pdf>

Eurostat. “Eurostat database, last updated November 17, 2014”. Luxembourg: Eurostat. Downloaded February 21, 2015 from http://appsso.eurostat.ec.europa.eu/nui/show.do?dataset=rd_e_gerdfund&lang=en.

Fogel, R.W. “Capitalism and Democracy in 2040: Forecasts and Speculations.”NBER Working Paper 13184. Washington, D.C.: National Bureau of Economic Research, 2007. <http://www.nber.org/papers/w13184>.

Fouré, J., A. Bénassy-Quéré and L. Fontagné. “The Great Shift: Macroeconomic Projections for the World Economy at the 2050 Horizon.” Paris: Centre D’études Prospectives et D’informations, Internationales. 2012. <http://www.cepii.fr/anglaisgraph/workpap/pdf/2012/wp2012-03.pdf>

Gokhberg, L. and L. Mindely. “Soviet R&D Resources: Basic Characteristics,” in S. Glaziev and C.M. Schneider, eds. Research and Development Management in the Transition to a Market Economy, Laxenburg, Austria: International Institute for Applied Systems Analysis, 1993.

Gokhberg, L., M.J. Peck, and J. Gács, eds. Russian Applied Research and Development: Its Problems and Its Promise. Laxenburg, Austria: International Institute for Applied Systems Analysis, 1997.

Goldman Sachs Global Economics Group. *BRICs and Beyond*. London: Goldman Sachs Group, Inc. 2007. <http://www.goldmansachs.com/our-thinking/topics/brics/brics-and-beyond-book-pdfs/brics-full-book.pdf>

GOSKOMSTAT (Central Statistical Bureau of the USSR). "National Economy of the USSR in 1980." Moscow: Finance and Statistics, 1981, p. 524.

GOSKOMSTAT (Central Statistical Bureau of the USSR). "National Economy of the USSR in 1982." Moscow: Finance and Statistics, 1983, pp. 522-523.

GOSKOMSTAT (Central Statistical Bureau of the USSR). "National Economy of the USSR in 1985." Moscow: Finance and Statistics, 1986, pp. 561-562.

GOSKOMSTAT (Central Statistical Bureau of the USSR). "National Economy of the USSR in 1988." Moscow: Finance and Statistics, 1989, pp. 626-627.

GOSKOMSTAT (Central Statistical Bureau of the USSR). "National Economy of the USSR in 1989." Moscow: Finance and Statistics, 1990.

GOSKOMSTAT (Central Statistical Bureau of the USSR). "National Economy of the USSR in 1990." Moscow: Finance and Statistics, 1991.OECD (Organisation for Economic Co-operation and Development). “OECD.StatMain Science and Technology Indicators. (last updated June 26, 2012).” Paris: OECD. Downloaded August 20, 2012 from http://stats.oecd.org/index.aspx?r=910468#.

Maddison, A. *Contours of the World Economy 1-2030 AD: Essays in Macro-Economic History*. Oxford: Oxford University Press, 2007.

OECD (Organisation for Economic Co-operation and Development). *Looking to 2060: Long-term Growth Prospects for the World*. Paris: Organisation for Economic Co-operation and Development, 2012. <www.oecd.org/eco/economicoutlookanalysisandforecasts/lookingto2060.htm> (Access Date: 12-5-12)

OECD (Organisation for Economic Co-operation and Development). "OECD.StatExtracts, last updated February 3rd, 2015". Paris: OECD. Accessed on February 21, 2015 from http://stats.oecd.org/index.aspx?r=281748.

PwC (PricewaterhouseCoopers). *The World in 2050: The Accelerating Shift of Global Economic Power: Challenges and Opportunities*. United Kingdom: PricewaterhouseCoopers, 2011. <www.pwc.com/en_GX/gx/world-2050/pdf/world-in-2050-jan-2011.pdf>

PwC (PricewaterhouseCoopers). *The World in 2050: Will the Shift in Global Economic Power Continue*? United Kingdom: PricewaterhouseCoopers, 2015. <www.pwc.com/gx/en/issues/the-economy/assets/world-in-2050-february-2015.pdf>

RICYT (Red de Indicadores de Ciencia y Technologia). "Indicators". Buenos Aires, Argentina: RICYT, 2015. Downloaded February 21, 2015 from http://db.ricyt.org/query/AR,BO,BR,CA,CL,CO,CR,CU,EC,ES,GT,HN,JM,MX,NI,PA,PE,PR,PT,PY,SV,TT,US,UY,VE,AL,IB/1990%2C2011/GASTOxPBI.

UNESCO-UIS (United Nations Educational, Scientific and Cultural Organization Institute for Statistics). "UIS.Stat". Montreal, Canada: UNESCO-UIS, 2015. Downloaded February 22nd 2015 from http://stats.uis.unesco.org/unesco/ReportFolders/ReportFolders.aspx?IF_ActivePath=P,54&IF_Language=eng. Last update July 2014.

UNESCO (United Nations Educational, Scientific and Cultural Organization). UNESCO Science Report 2005. Paris: UNESCO, 2005.

UNESCO-UIS (United Nations Educational, Scientific and Cultural Organization-Institute for Statistics). "Statistical tables from the 1999 UNESCO Statistical Yearbook." Montreal, Canada: UNESCO-UIS, 1999. Downloaded July 12, 2005 from http://www.uis.unesco.org/Library/Documents/Historic_S-T_data.pdf.

UNESCO (United Nations Educational, Scientific and Cultural Organization). World Science Report 1998. Paris: UNESCO, 1998.

UNESCO (United Nations Educational, Scientific and Cultural Organization). World Science Report 1996. Paris: UNESCO, 1996.

UNESCO (United Nations Educational, Scientific and Cultural Organization). World Science Report 1993. Paris: UNESCO, 1993.

United States Department of Agriculture, Economic Research Service. "International Macroeconomic Data Set." Washington, D.C.: United States Department of Agriculture, 2012. <http://www.ers.usda.gov/data-products/international-macroeconomic-data-set.aspx> (Access Date: 12-7-12)

Ward, K. *The World in 2050: Quantifying the Shift in Global Economy.* London: HSBC Global Economics, 2011. <http://www.hsbc.com/~/media/HSBC-com/about-hsbc/in-the-future/pdfs/120508-the-world-in-2050.ashx>

World Bank. “World Development Indicators, last update January 30, 2015.” Washington, DC: World Bank. Downloaded February 22, 2015 from http://data.worldbank.org/data-catalog/world-development-indicators.

Figures and Tables

Table A1: Primary data sources for GERD intensity estimates

	File				Data	
Agency	Name	Countries covered	Years covered	File accessed	File last updated
**Primary Sources**					
Eurostat (2015)	Total intramural R&D expenditure (GERD) by sectors of performance and source of funds	37	1981-2013	2-21-2015	11-17-2014
OECD (2015)	Main Science and Technology Indicators (MSTI database)	41	1981-2014	2-21-2015	02-03-2015
RICYT (2015)	Expenditure on S&T as a percentage of GDP	25	1990-2011	2-21-2015	NR
UNESCO (2015)	Total intramural R&D expenditure (GERD) by sectors of performance and source of funds	125	1996-2012	2-22-2015	July 2014
World Bank (2015)		133	1996-2012	2-22-2015	1-30-2015
**Secondary Sources**					
UNESCO (2005)		59	1997-2004	NA	NA
UNESCO(1999)		91	1980-1997	07-12-2005	NR
UNESCO (1998)		60	1980-1996	NA	NA
UNESCO (1996)		41	1980-1993	NA	NA
UNESCO (1993)		72	1980-1992	NA	NA

Notes: NA indicates not applicable. NR indicates not reported. The data obtained from UNESCO (1998, 1996 and 1993) were manually extracted from hard copies of the respective published volumes.

Table A2: Regional classifications for InSTePP global GERD database

WB Country	Region	WB Country	Region	WB Country	Region
Afghanistan	Asia&Pacific	Grenada	LAC	Panama	LAC
Albania	EE&FSU	Guatemala	LAC	Papua New Guinea	Asia&Pacific
Algeria	MENA	Guinea	SSA	Paraguay	LAC
Andorra	High Income	Guinea-Bissau	SSA	Peru	LAC
Angola	SSA	Guyana	LAC	Philippines	Asia&Pacific
Antigua and Barbuda	High Income	Haiti	LAC	Poland	EE&FSU
Argentina	LAC	Honduras	LAC	Portugal	High Income
Armenia	EE&FSU	Hong Kong SAR, China	High Income	Puerto Rico	High Income
Australia	High Income	Hungary	EE&FSU	Qatar	High Income
Austria	High Income	Iceland	High Income	Romania	EE&FSU
Azerbaijan	EE&FSU	India	Asia&Pacific	Russian Federation	EE&FSU
Bahamas, The	High Income	Indonesia	Asia&Pacific	Rwanda	SSA
Bahrain	High Income	Iran, Islamic Rep.	MENA	Samoa	Asia&Pacific
Bangladesh	Asia&Pacific	Iraq	MENA	San Marino	High Income
Barbados	High Income	Ireland	High Income	Sao Tome and Principe	SSA
Belarus	EE&FSU	Israel	High Income	Saudi Arabia	High Income
Belgium	High Income	Italy	High Income	Senegal	SSA
Belize	LAC	Jamaica	LAC	Serbia	EE&FSU
Benin	SSA	Japan	High Income	Seychelles	SSA
Bermuda	High Income	Jordan	MENA	Sierra Leone	SSA
Bhutan	Asia&Pacific	Kazakhstan	EE&FSU	Singapore	High Income
Bolivia	LAC	Kenya	SSA	Slovak Republic	EE&FSU
Bosnia and Herzegovina	EE&FSU	Kiribati	Asia&Pacific	Slovenia	EE&FSU
Botswana	SSA	Korea, Rep.	High Income	Solomon Islands	Asia&Pacific
Brazil	LAC	Kosovo	EE&FSU		
Brunei Darussalam	High Income	Kuwait	High Income	South Africa	SSA
Bulgaria	EE&FSU	Kyrgyz Republic	EE&FSU	Soviet Union	EE&FSU
Burkina Faso	SSA	Lao PDR	Asia&Pacific	Spain	High Income
Burundi	SSA	Latvia	EE&FSU	Sri Lanka	Asia&Pacific
Cambodia	Asia&Pacific	Lebanon	MENA	St. Kitts and Nevis	High Income
Cameroon	SSA	Lesotho	SSA	St. Lucia	LAC
Canada	High Income	Liberia	SSA	St. Vincent and the Grenadines	LAC
Cape Verde	SSA	Libya	MENA	Sudan	SSA
Central African Republic	SSA	Liechtenstein	High Income	Suriname	LAC
Chad	SSA	Lithuania	EE&FSU	Swaziland	SSA

Table A2: Regional classifications for InSTePP global GERD database (continued)

WB Country	Region	WB Country	Region	WB Country	Region
Chile	High Income			Sweden	High Income
China	Asia&Pacific	Macao SAR, China	High Income	Switzerland	High Income
Colombia	LAC	Macedonia, FYR	EE&FSU	Syrian Arab Republic	MENA
Comoros	SSA	Madagascar	SSA	Taiwan	High Income
Congo, Dem. Rep.	SSA	Malawi	SSA	Tajikistan	EE&FSU
Congo, Rep.	SSA	Malaysia	Asia&Pacific	Tanzania	SSA
Costa Rica	LAC	Maldives	Asia&Pacific	Thailand	Asia&Pacific
Cote d'Ivoire	SSA	Mali	SSA	Timor-Leste	Asia&Pacific
Croatia	EE&FSU	Malta	High Income	Togo	SSA
		Marshall Islands	Asia&Pacific	Tonga	Asia&Pacific
Cyprus	High Income	Mauritania	SSA	Trinidad and Tobago	High Income
Czech Republic	EE&FSU	Mauritius	SSA	Tunisia	MENA
Denmark	High Income	Mexico	LAC	Turkey	MENA
Djibouti	SSA	Micronesia, Fed. Sts.	Asia&Pacific	Turkmenistan	EE&FSU
Dominica	LAC	Moldova	EE&FSU	Turks and Caicos Islands	High Income
Dominican Republic	LAC	Monaco	High Income		
Ecuador	LAC	Mongolia	Asia&Pacific	Uganda	SSA
Egypt, Arab Rep.	MENA	Montenegro	EE&FSU	Ukraine	EE&FSU
El Salvador	LAC	Morocco	MENA	United Arab Emirates	High Income
Equatorial Guinea	High Income	Mozambique	SSA	United Kingdom	High Income
Eritrea	SSA	Myanmar	Asia&Pacific	United States	High Income
Estonia	EE&FSU	Namibia	SSA	Uruguay	High Income
Ethiopia	SSA	Nepal	Asia&Pacific	Uzbekistan	EE&FSU
Fiji	Asia&Pacific	Netherlands	High Income	Vanuatu	Asia&Pacific
Finland	High Income	New Zealand	High Income	Venezuela, RB	LAC
France	High Income	Nicaragua	LAC	Vietnam	Asia&Pacific
Gabon	SSA	Niger	SSA	West Bank and Gaza	MENA
Gambia, The	SSA	Nigeria	SSA	Yemen, Rep.	MENA
Georgia	EE&FSU	Norway	High Income	Zambia	SSA
Germany	High Income	Oman	High Income	Zimbabwe	SSA
Ghana	SSA	Pakistan	Asia&Pacific		
Greece	High Income	Palau	Asia&Pacific		

Notes: EE=Eastern Europe; FSU=Former Soviet Union; LAC= Latin America and Caribbean; MENA=Middle East and North Africa; SSA=Sub-Saharan Africa.

Figure A1: Indexed Decomposition of the Sources of GERD Growth, 1980-2013

a. By income classes

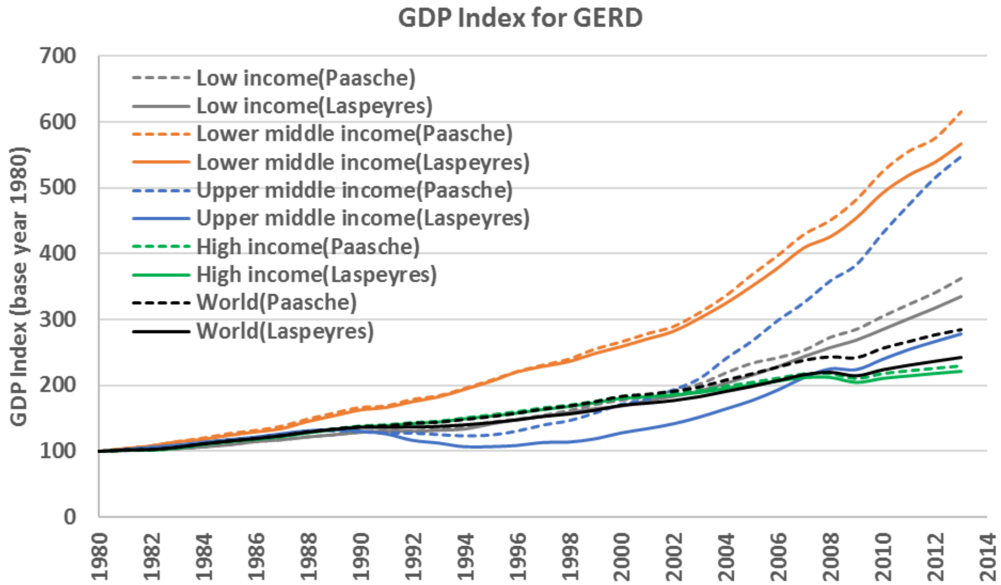

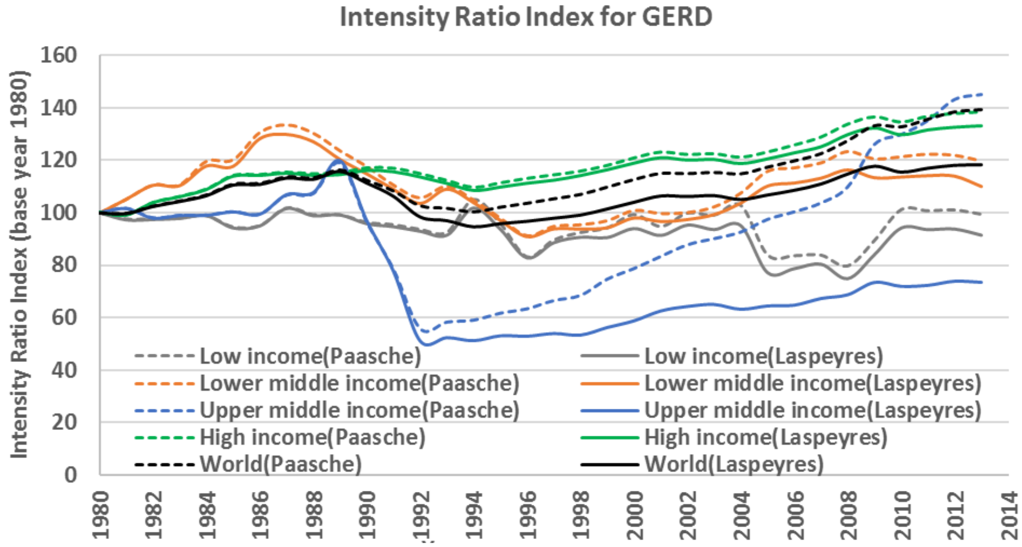

b. By geographic regions

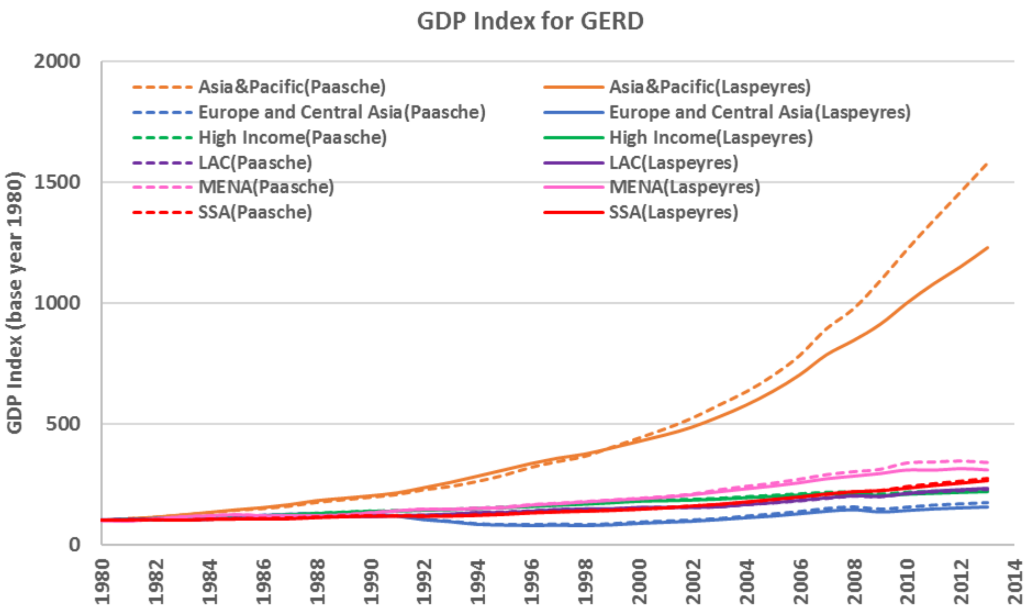

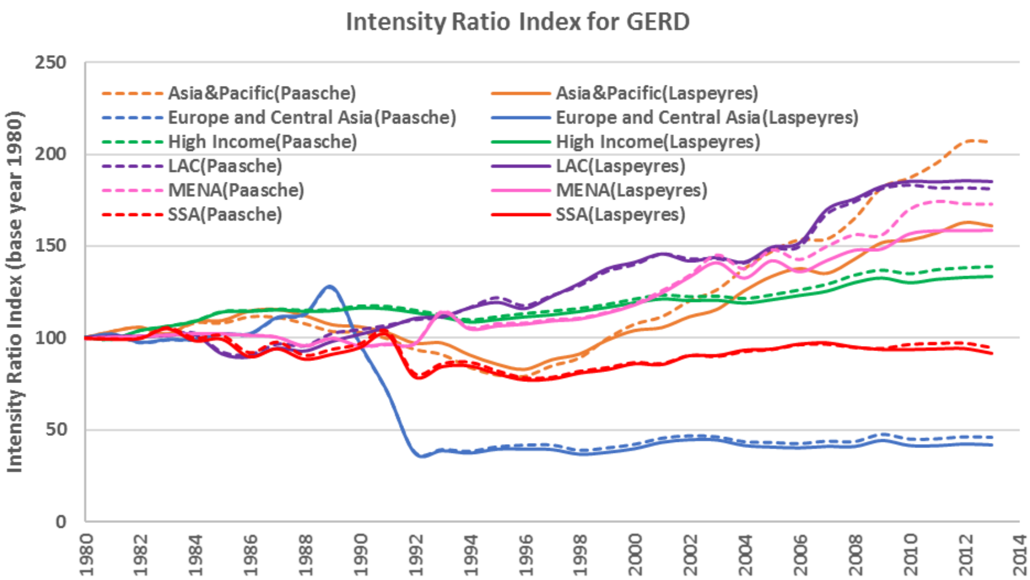

Source: Estimated by authors.

Notes: These figures show the decomposition of sources of GERD growth as GDP Index and Intensity Ratio Index with a base year of 1980. Countries are grouped by both income classes (panel a) and geographic regions (panel b).

Figure A2: Logarithmic Difference Decomposition of the Sources of GERD Growth, 1980-2013

a. By income classes

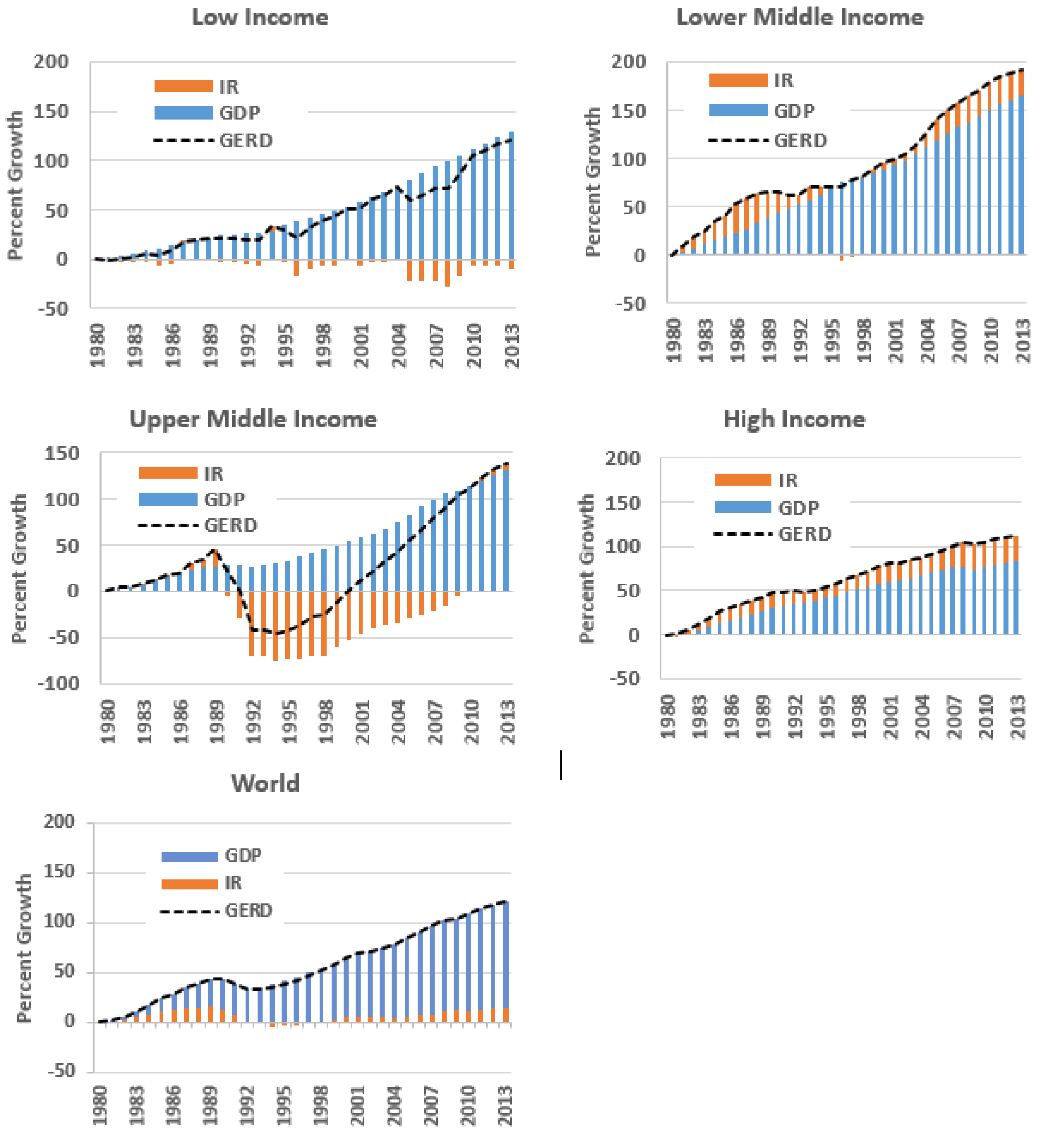

b. By geographic regions

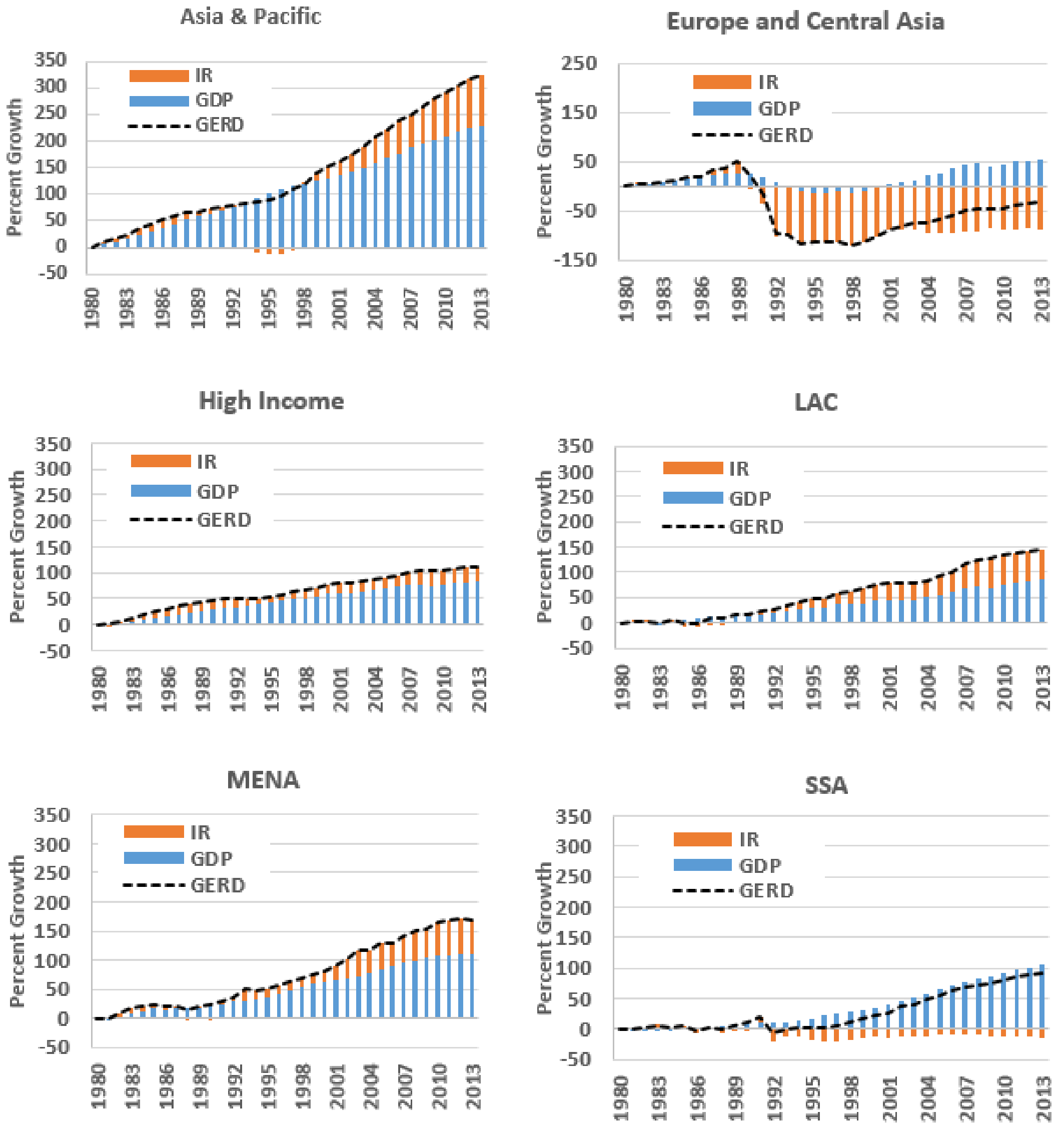

Source: Estimated by authors.

Notes: These figures show the logarithmic difference decomposition of the sources of GERD growth into intensity ratio (IR) growth and GDP growth with a base year of 1980. Countries are grouped by both income classes (panel a) and geographic regions (panel b).

Figure A3: GERD Intensity, 2013

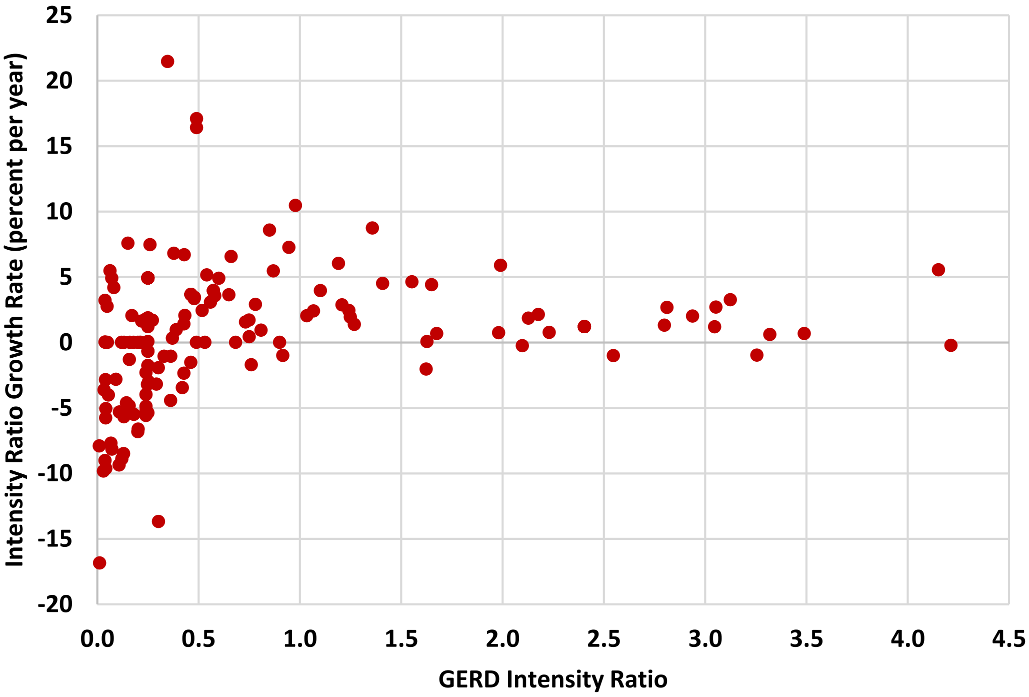

Source: Estimated by authors.

Notes: The figure shows each country's GERD intensity ratio on the horizontal axis and the 2003-2013 continuous intensity growth rate on the vertical axis.

Figure A4: GERD Intensity Trends, 1980-2050

Panel a: Simple average

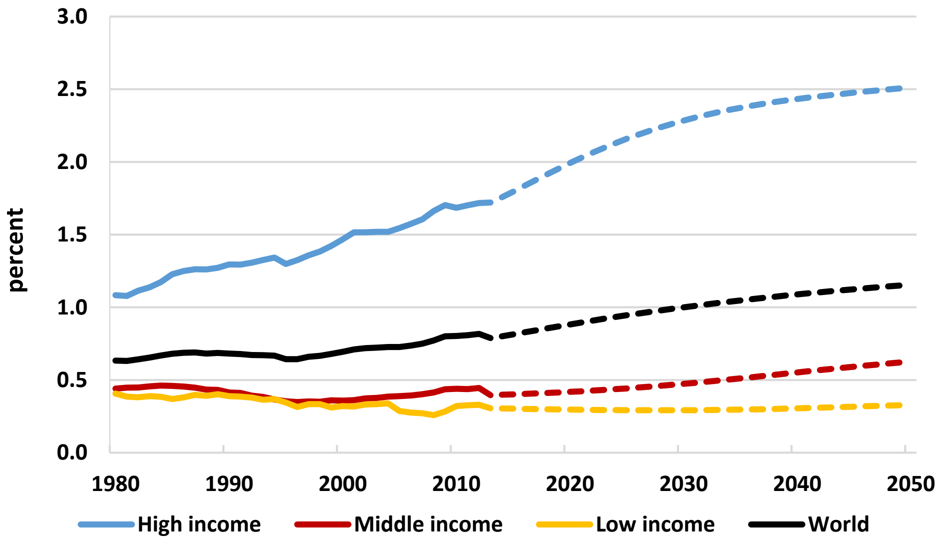

Panel b: Weighted average

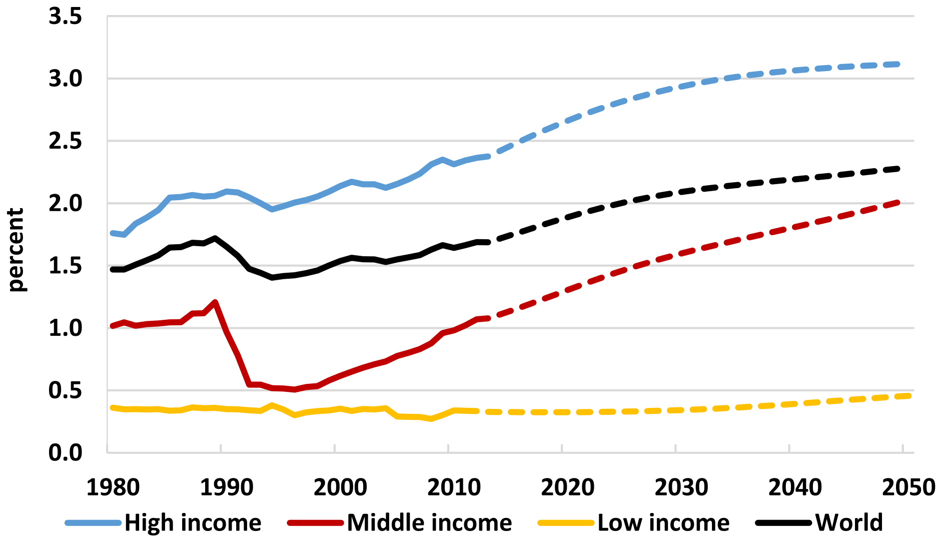

Source: Developed by authors.

Notes: Solid lines represent historical estimates; dashed lines represent projected data.

Figure A5: GERD Intensity Trends: Income Averages and Country Level Dispersions, 1980-2013

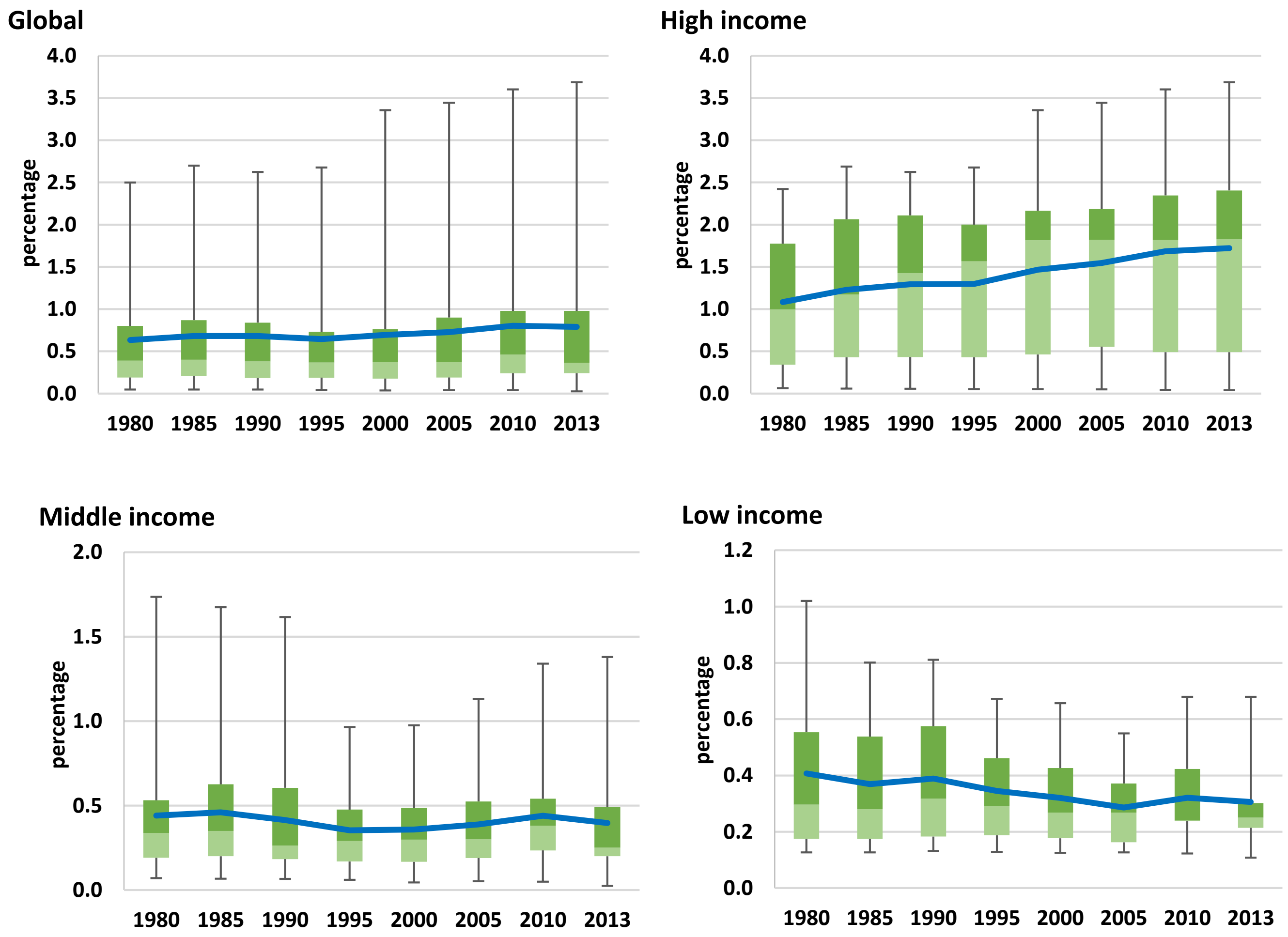

Source: Developed by authors.

Notes: The top and bottom endpoints of the whisker represent the simple average GERD intensity for the top 5 and bottom 5 countries respectively; the bottom and top of the box are the first and third quartiles; and the segment where the dark and light green area meet represent the median. The blue colored trend line is the simple average research intensity for each income class and the world.

Table A3: Average Rates of Growth of Country-Level GERD Intensities, 1980-2013

	Countries	
	Number	Share
	(count)	(*percentage*)
Equal or less than 0%	71	40.6
Between 0% and 1%	49	28.0
Greater than 1%	55	31.4
**Total**	**175**	**100.0**

Source: Compiled by authors.

Notes: Underlying growth rates calculated by least squares regression method.

Table A4: Historical GERD Growth Rates and GERD Intensity Ratios and Growth Rates, 1980-2013

	GERD intensity ratios						GERD Intensity growth rates						GERD growth rates					
Period	Mean	Median	Min	Max	25th	75th	Mean	Median	Min	Max	25th	75th	Mean	Median	Min	Max	25th	75th
	(percent)						(percent per year)											
**High income**																		
1980s	1.19	1.16	0.04	2.76	0.42	1.97	1.76	1.94	-9.49	13.87	0.00	3.15	5.1	5.0	-9.1	25.3	2.4	7.5
1990s	1.34	1.55	0.04	3.11	0.43	2.05	0.69	0.24	-9.10	9.52	0.00	2.86	4.9	4.5	-7.2	24.0	2.2	6.5
2000s	1.61	1.88	0.03	4.19	0.49	2.29	1.56	0.96	-10.04	16.36	0.04	2.89	4.5	3.3	-5.9	15.7	1.8	7.2
1980-2013	1.46	1.76	0.04	3.57	0.48	2.17	1.20	0.73	-8.45	5.66	0.01	3.17	4.9	4.2	-5.6	16.5	2.7	6.9
**Middle income**																		
1980s	0.45	0.33	0.04	2.76	0.22	0.57	-0.73	0.00	-10.64	11.34	-2.16	0.50	2.5	1.9	-11.4	13.3	0.0	5.7
1990s	0.37	0.30	0.04	1.24	0.17	0.50	-0.98	-0.67	-17.87	10.39	-3.28	1.70	2.5	2.5	-14.8	13.7	0.3	6.0
2000s	0.41	0.33	0.04	1.59	0.21	0.51	0.99	1.81	-11.63	19.93	-2.32	3.62	5.5	5.5	-8.5	25.3	1.4	8.9
1980-2013	0.41	0.35	0.04	1.59	0.21	0.54	-0.29	0.01	-5.33	7.58	-1.57	1.10	3.4	2.8	-3.7	13.7	1.3	5.5
**Low income**																		
1980s	0.39	0.30	0.05	1.12	0.18	0.54	0.65	0.00	-6.40	12.02	-0.74	0.50	3.0	3.1	-6.2	15.4	-0.6	4.6
1990s	0.35	0.33	0.05	0.78	0.18	0.50	-1.68	-0.32	-14.87	10.17	-3.91	0.00	0.5	1.1	-17.1	15.6	-3.5	5.1
2000s	0.31	0.25	0.05	0.74	0.20	0.40	-0.16	1.50	-12.22	4.45	-2.92	3.08	5.0	5.2	-8.7	14.6	1.8	8.0
1980-2013	0.34	0.30	0.05	0.75	0.19	0.46	-0.65	0.01	-5.56	4.00	-2.66	0.59	2.4	2.3	-4.6	11.3	-0.2	5.0
**World**																		
1980s	0.67	0.42	0.04	2.76	0.21	0.82	0.29	0.00	-10.64	13.87	-1.13	1.94	3.4	3.3	-11.4	25.3	0.7	6.8
1990s	0.67	0.37	0.04	3.11	0.18	0.83	-0.59	0.00	-17.87	10.39	-2.08	1.70	2.9	3.4	-17.1	24.0	0.7	6.2
2000s	0.76	0.40	0.03	4.19	0.21	0.92	0.96	1.44	-12.22	19.93	-1.13	3.08	5.1	4.9	-8.7	25.3	1.7	8.1
1980-2013	0.72	0.41	0.04	3.57	0.21	0.84	0.11	0.23	-8.45	7.58	-1.04	1.31	3.7	3.3	-5.6	16.5	1.5	5.8

Source: Compiled by authors.

Notes: 1980s represents the period 1980 to 1990: 1990s, the period 1990 to 2000; and 2000s the period 2000 to 2013. Growth rates calculated by least squares regression method.

Table A5: Sources for GDP Projections for Midline, Low and High Estimates, 2015-2050

Source	Midline		Low Estimate		High Estimate	
	2015-2030	2031-2050	2015-2030	2031-2050	2015-2030	2031-2050
			(percentage)			
Carnegie (2010)	0	0	0.1	0	0.5	1.5
CEPII (2012), country-specific	65.8	65.8	8.8	42.7	37.3	48.9
CEPII (2012), regional average	25.4	25.4	5.8	17.3	16.4	22.1
Fogel (2007)	0	0	15.7	7.6	15.5	6.8
Goldman Sachs (2007)	0	0	0.4	0.1	4.5	5.3
HSBC (2011)	0	0	4.6	1.6	2.6	6.2
Maddison (2007)	0	0	51.7	20.8	10.6	5.1
OECD (2012)	0	0	1.6	9.8	2.0	1.8
PwC (2011)	0	0	0.1	0.1	2.2	2.4
PwC (2015)	8.8	8.8	0	0	0	0
USDA, ERS (2012)	0	0	11.2	0	8.3	0

Source: Compiled by authors.

Notes: "Percentage" indicates the share of observations sourced from the respective sources. "CEPII (2012), country-specific" refers to country estimates derived directly from the source. "CEPII (2012), regional average" refers to cases where country-specific estimates were not reported so that the average of the annual projected growth rate for each country's respective region was used as a proxy for the country-specific growth rate (with the caveat that Brazil, China, India, Nigeria and South Africa were omitted from each of their respective regions when forming regional average growth rates).

Table A6: Past and projected rates of GDP growth, 1980-2050

	1980-2013 period				
	1980-1990	1990-2000	2000-2013	1980-2013	2013-2050
	(percent per year)				
China	10.3	10.6	10.4	10.0	3.5
India	5.8	6.0	7.6	6.2	4.9
Asia&Pacific excl. China and India	5.1	4.5	5.3	5.0	4.4
Asia&Pacific	6.7	7.1	8.3	7.2	4.1
Japan	4.6	1.0	0.7	1.9	1.4
United States	3.7	3.6	1.7	2.9	1.6
High income excl. USA and Japan	2.7	2.9	2.1	2.7	1.8
High Income	3.3	2.9	1.8	2.7	1.7
EE&FSU	2.7	-2.9	4.5	1.0	2.1
Brazil	2.8	2.8	3.6	2.7	2.8
LAC excl. Brazil	1.2	3.0	3.8	2.7	3.4
LAC	1.8	2.9	3.7	2.7	3.2
MENA	3.2	4.0	4.4	3.8	4.2
SSA	1.2	2.5	5.7	3.4	5.5
**World**	3.3	3.1	3.9	3.4	3.1

Source: Estimated by authors based on the ”midline" sources summarized in Tables A1 and A7.

Notes: All nominal GDP series were first deflated with the respective country-specific implicit GDP deflator and the growth rates were then calculated using the least-squares method.

Figure A6: GDP and GDP Per Capita Projections

Panel a: GDP projection

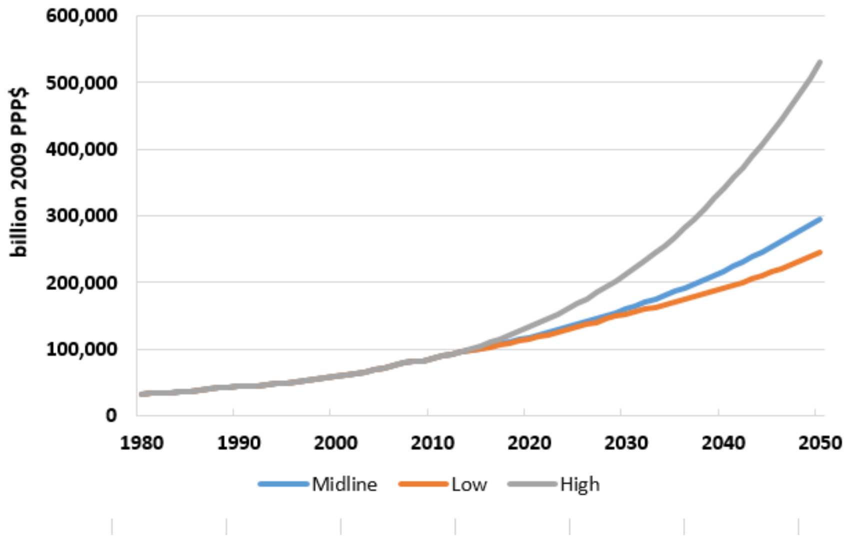

Panel b: GDP per capita projection

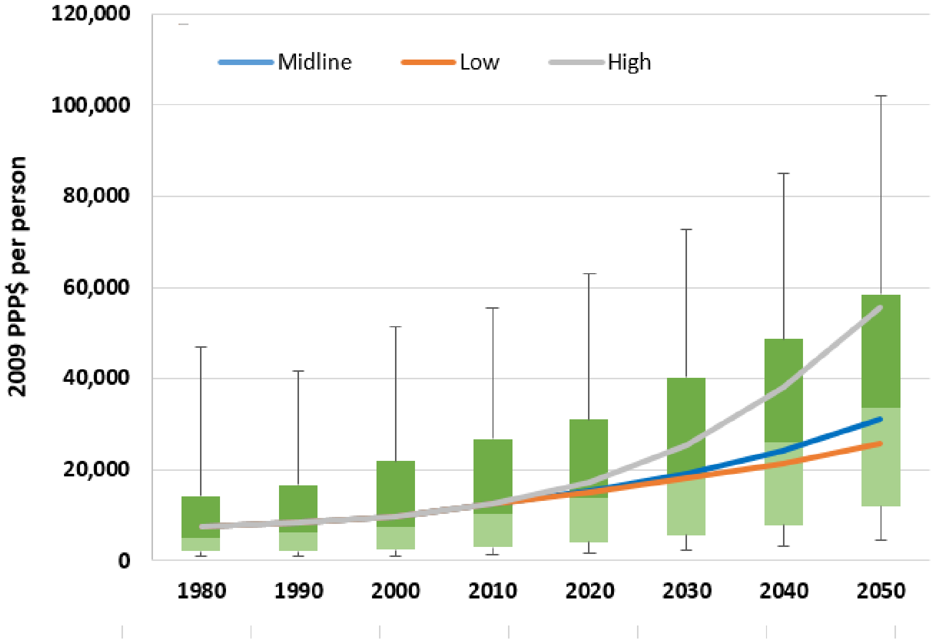

Source: Estimated by authors based on sources noted in the body of Table A7.

Notes: The top and bottom endpoints of the whisker represent the simple average of GDP per capita for the top 30 and bottom 30 countries; the bottom and top of the box are the first and third quartiles. The segment where the dark and light green areas meet represent the median. The trend lines of GDP per capita (midline, low, and high) are weighted averages.

Table A7: GERD Projections Details—Midline and its Variants, with 2050 Endpoints

Scenario	Global GERD	Global GERD Intensity	Notes
	(trillion 2009 $Int)	(*percentage*)	
**Dynamic Intensity Ratios**			
Midline	6.76	2.29	Projected midline GDP (Table A7)
Low variant	6.02	2.46	Projected low variant GDP (Table A7)
High variant	11.13	2.10	Projected high variant GDP (Table A7)
**Static Intensity Ratios**			
Constant country	4.02	1.36	Country-specific intensity ratios held at 2013 levels and applied to midline GDP (causing the global intensity to decrease over time).
Constant global	4.98	1.69	Global intensity ratio held at 2013 level (1.69 percent) to derive global GERD, which was then allocated to countries in proportion to their midline GDP projection.
